# More continuity than change following the Black Death epidemic in medieval Cambridge

**DOI:** 10.1038/s41598-025-18437-5

**Published:** 2025-09-29

**Authors:** John Robb, Jenna M. Dittmar, Sarah A. Inskip, Alice K. Rose, Piers D. Mitchell, Tamsin C. O’Connell, Mary Price, Craig Cessford

**Affiliations:** 1 University of Cambridge, Cambridge, UK; 2 Edward Via College of Osteopathic Medicine, Blacksburg, USA; 3 University of Leicester, Leicester, UK; 4 University of York, York, UK; 5 Independent Researcher, Ellesmere Port, UK

**Keywords:** Public health, Socioeconomic scenarios

## Abstract

The Black Death epidemic of *Yersinia pestis* (1347-50 CE) killed about half the population of England, and many historical changes have been ascribed to it. But we still know surprisingly little about how the epidemic actually affected people’s daily lives. This study reports results from a broad bioarchaeological study, with 18 skeletal and molecular indicators from 336 adults from Cambridge, England (940–1561 CE). Results reveal two major findings. First, although the epidemic killed millions, no dramatic changes in skeletal indicators of health and lifestyle are directly ascribable to the epidemic. Most indicators either remained stable, or changed in response to other known historical trends. Traumatic though the plague was, it did not transform medieval people’s health and lifeways radically. Secondly, there were important long-term health trends beginning before the Black Death, notably a decline in stature. This may be due to a general 14th -century environmental and economic deterioration which left the population more vulnerable to novel epidemic diseases.

**Supplementary Information:**

The online version contains supplementary material available at 10.1038/s41598-025-18437-5.

## Introduction

Particularly in light of the Covid-19 pandemic of 2019-23, there has been increased attention to epidemics and their social and historical consequences. However, the historical consequences of epidemics are surprisingly ambiguous. The influenza pandemic of 1918-20, for instance, killed more people globally than the First World War did, but its historical effects seem to have been surprisingly transient compared to many less lethal social, political, economic and technological developments of its time^1^. The same may be true for the late 19th century Third Plague Pandemic of *Yersinia pestis* in India and China, and the significance of other historical plagues such as the First Pandemic remains controversial^2,3^.

The Black Death, the first several years of the second pandemic of *Yersinia pestis* (bubonic plague), is the best-documented and studied pre-modern epidemic in world history. As such, it provides us with the best opportunity to explore this question. During the Black Death, *Y. pestis* swept through Asia, Africa, and Europe in 1347-53, killing millions. Here we focus upon its effects in Europe. The epidemic’s direct effect is undoubted: the death toll reached 50% or higher in some areas; it initiated the Second Pandemic, a 300-year period in which deadly plague epidemics recurred every 10–20 years^4^. Some effects are clearly documented historically and archaeologically, particularly in England. The demographic effects were catastrophic; the population of England dropped from around 5 million to about half that. The country stagnated demographically, only regaining its former population level 2–3 centuries later^5^. The changing balance of people and resources had knock-on effects. In the landscape, settlements were abandoned and fields turned to pasture, which required less labour and which powered England’s late medieval wealth-creating wool trade. Socially, wages rose as the labour supply was reduced, and workers became less tied to estates and more independently mobile^6,7^. How to interpret such changes has shifted over the years^8^. A traditional narrative, still common in popular literature, held that the Black Death transformed medieval society, initiating the ”Golden Age of the English Labourer” and a social and political shift to the modern world^9^. However, most historians take a more nuanced view. There was arguably much more continuity than change in social and political life. Both in England and throughout Europe generally, there was little challenge to political stability and entrenched social structures; no social groups, ranks or identities vanished or appeared, no technologies or bodies of knowledge were lost, and religious reformulation was subtle and gradual, not dramatic. Many historians have seen the plague as hastening and amplifying changes which were happening anyway (for example, the transition from serfs tied to estates to free labour)^10,11^. Both socially and ecologically, the Black Death can be seen as a turning point, but also must be understood as part of longer-term social contexts. For example, Western Europe may have been overpopulated and experiencing climatic deterioration and decreasing harvests for up to a century before the plague^12^. The Great Famine of 1315-20 which killed 10–15% of the English population may have been the opening salvo of a century of population rebalancing. Indeed, as a consequence of such long-term climatic and demographic trends, when the epidemic struck, it may have hit a malnourished, vulnerable population^13,14^.

However, the plague’s long-term effects on health and lifestyle remain elusive. Bioarchaeology can add significantly to such debates by providing direct evidence about the effects of plague on diet, susceptibility to disease, lifespan, and other aspects of social life. For example, if wages increased, did people actually consume a better diet? With a lower population, were they less likely to suffer from infectious diseases? Did they grow more successfully and live longer? Bioarchaeology provides the most direct way to answer such questions. Many bioarchaeological studies of the Black Death have studied people who actually died during the pandemic to answer questions such as whether the plague killed randomly or selectively^15^. Studies comparing living standards in pre-plague and post-plague groups to examine how the epidemic changed human lives are only starting to appear^16–20^. Bioarchaeological studies can address important hypotheses about the historic consequences of the epidemic. For example, improved diet may be visible isotopically; improved childhood health should result in higher adult stature (Table 1).

**Table 1:** Possible historical consequences of the black death and their potential skeletal/ molecular evidence.

Documented consequence of epidemic	Possible effects on lifestyle	Possible bioarchaeological evidence
Drop in population; lower population density	Less crowding, in housing and in towns Less infectious disease	Lowered prevalence of infectious diseases Higher age at death
Increase in wages and consumption	Better nutrition, better clothing, better housing	Improved childhood environment, fewer deficiency diseases Increased δ^15^N (and potentially δ^13^C) values in all groups Increased stature
Shift in land use towards pastoralism	Increased availability of meat and dairy products	Increased δ^15^N values
Increased mobility	People moving between rural work, and between rural areas and towns	Increased diversity in 87Sr/86Sr isotope ratios Increased genetic diversity within local area

This study, focusing upon medieval Cambridge, develops this line of research in two innovative ways. First, rather than focusing upon a one or a few dimensions of health and lifestyle such as stature, longevity, or diet, it uses a broad, multidisciplinary repertory of 18 skeletal and molecular indicators of illness, diet and activity (see “Methods” below, and Table 2) to provide a broad picture of how human lives may have changed. Bioarchaeological data address only physical health rather than mental or spiritual health, and there are many aspects of health and lifestyle which skeletal and molecular indicators do not address, but nevertheless studies such as this can add new depth to understanding of health and lifestyle. Secondly, using well-contextualised samples, we can confront the complexity of medieval life. For example, medieval Cambridge was socially differentiated, with important health inequalities^21,22^. Rather than assuming that available samples must be representative of their time, we investigate how much apparent changes may be due to the social backgrounds of the samples. Similarly, if a change is observed following the Black Death, we cannot assume that must have been caused by the Black Death. Instead, we investigate the extent to which observed changes actually coincide chronologically with the epidemic or may derive from other social changes.

**Table 2:** Bioarchaeological data collected.

Category	Data collected and references^21^
Demographic estimation	Sex estimation: osteology^23,24^; genetic estimation^25^. Age at death estimation: ^26–32^). Broad age categories are used to accommodate known imprecision in adult age at death estimates, particularly for older individuals
Linear enamel hypoplasia	Anterior surfaces of teeth were examined under strong oblique light. LEH was recorded as “present” in a dentition when the lesion was palpable and more than one tooth was affected
Cribra orbitalia	Recorded as “present” when areas of porosity were observed on the superior surface of at least one orbit which was more than 50% complete (types 2–5, Stuart-Macadam 1991:109)^33^. Both active and healed/ residual lesions were counted as “present”
Vitamin D deficiency	Skeletal signs such as bowed limb bones, flaring of the rib ends and metaphyses; diagnosed only when tibia were present and observable^34,35^
Maxillary sinusitis	Active or healed new reactive bone formation in maxillary sinuses^36^
New bone formation	Presence/ absence, location and type (woven, lamellar) of subperiosteal new bone formation; recorded as present/ absent for all skeletal elements. Scored as absent if not observed anywhere in skeleton. Ribs were recorded separately (see below)
Respiratory infection	Presence of subperiosteal new bone formation on the visceral surface of ribs, indicating pleural inflammation
Tuberculosis	Pathognomic signs of tuberculosis or substantial destructive remodelling of ribs and/or vertebra^37,38^. Considered “present” when an individual presented at least two of these lesions; (1) subperiosteal new bone formation on the visceral surfaces of the ribs, (2) destructive remodelling or lytic lesions on the visceral surfaces of the ribs, sternum, manubrium, anterior surface of the sacrum, os coxae, and/or cranium, (3) joint destruction^39–42^
DISH (Diffuse Idiopatic Skeletal Hyperostosis)	Recorded only when majority of thoracic and lumbar vertebrae were present. As per Rogers and Waldron^43^
Hallux valgus	Diagnostic signs of hallux valgus in the first metatarsal^44,45^
Osteoarthritis	As per Waldron (p. 34)^46^; counted as “present” if osteoarthritis observed in locations other than the spinal column
Schmorl’s nodes	Presence of resorptive lesions > 1 mm in depth on upper and/or lower surfaces of vertebral bodies
Trauma and cranial trauma	Healed or unhealed trauma anywhere in skeleton. Scored as absent if not observed anywhere in the skeleton^47^
Cranial trauma	Presence of healed or unhealed cranial trauma^47^
Adult stature	Estimated using Trotter and Gleser “white” equations, Table 19.2^48^
Dietary isotopes	δ^13^C and δ^15^N measured in dentine samples for childhood diet and in rib bone samples for diet in last decades of life (see methods listed above)

In 1200, Cambridge was a small but rapidly growing town of around 2000 people^49^. The university was founded around 1209, one of only two medieval universities in England. By 1348, the town had reached its peak medieval population, a medium-sized town of 3000–5000 people, including 500–700 male clerics belonging to monasteries, friaries and the university. The plague struck Cambridge as hard as anywhere in England, killing hundreds; some neighbourhoods were almost abandoned. After the epidemic, it slowly regained population. The mid/late 1300s and 1400s were marked by rapid expansion of the university, particularly with the foundation of new colleges under aristocratic and royal patronage. The later medieval university both increased the clerical presence in the city and brought in wealth, creating a reliable demand for goods and services that provided an economic base beyond farming and regional trade^50^.

Throughout this period, Cambridge had a diverse social landscape. Most people (perhaps 80–90% of the population) were ordinary working people on a spectrum from poor to prosperous. Almost all worked with their hands, whether in town or agriculturally in the fields around Cambridge. They varied in skills and capital from precarious casual labourers to trained craftspeople and smallholders. Secondly, while wealthy people, gentry and aristocracy were statistically insignificant, the poor were numerous, including people unable to work and/or living in severe poverty. The poor most visible historically were those living upon charity in formal institutions such as the Hospital of St. John the Evangelist (perhaps 1% of the population). A third social group consisted of religious professionals—clergy, monks, friars and nuns. In most towns approximately 2–5% of adult males would have been in this group; in Cambridge they were substantially more numerous (perhaps 15% of adult males) due to both the university and the presence of major religious houses. As detailed below (“Methods”), each of these groups had distinct patterns of health, diet and lifestyle which are visible skeletally and molecularly^21,22^.

Three hypotheses are possible:

1. The people of Cambridge experienced no significant change in health and lifestyle between the 13th and 15th centuries.
2. There was a coherent suite of changes in health and lifestyle which began after 1349 and can plausibly be related to known historical effects of the Black Death.
3. There were some changes in health and lifestyle in later medieval times, but other things also show continuity, the observed changes may not coincide with the timing of the Black Death, and the observed changes can plausibly be related to other known historical developments.

## Results

Examining bioarchaeological data for historical change reveals several important findings. The first is simply that many aspects of human biology do not display significant changes between 1200 and 1500 CE. One of the most significant, human genetics, has already been reported^51^. Ancient DNA extracted from samples drawn from these groups showed no discernible differences from more recent historical and modern people living in East Anglia, demonstrating substantial continuity of population. Moreover, SNPs specifically related to disease resistance did not show significant historical change, either in comparison with modern populations or in comparison of groups before and after the Black Death. This suggests that neither the Black Death or the repeated episodes of plague following it (the Second Pandemic, which lasted in England until 1665-6 CE) exerted an important selection effect upon the human genome.

**Fig. 1:**
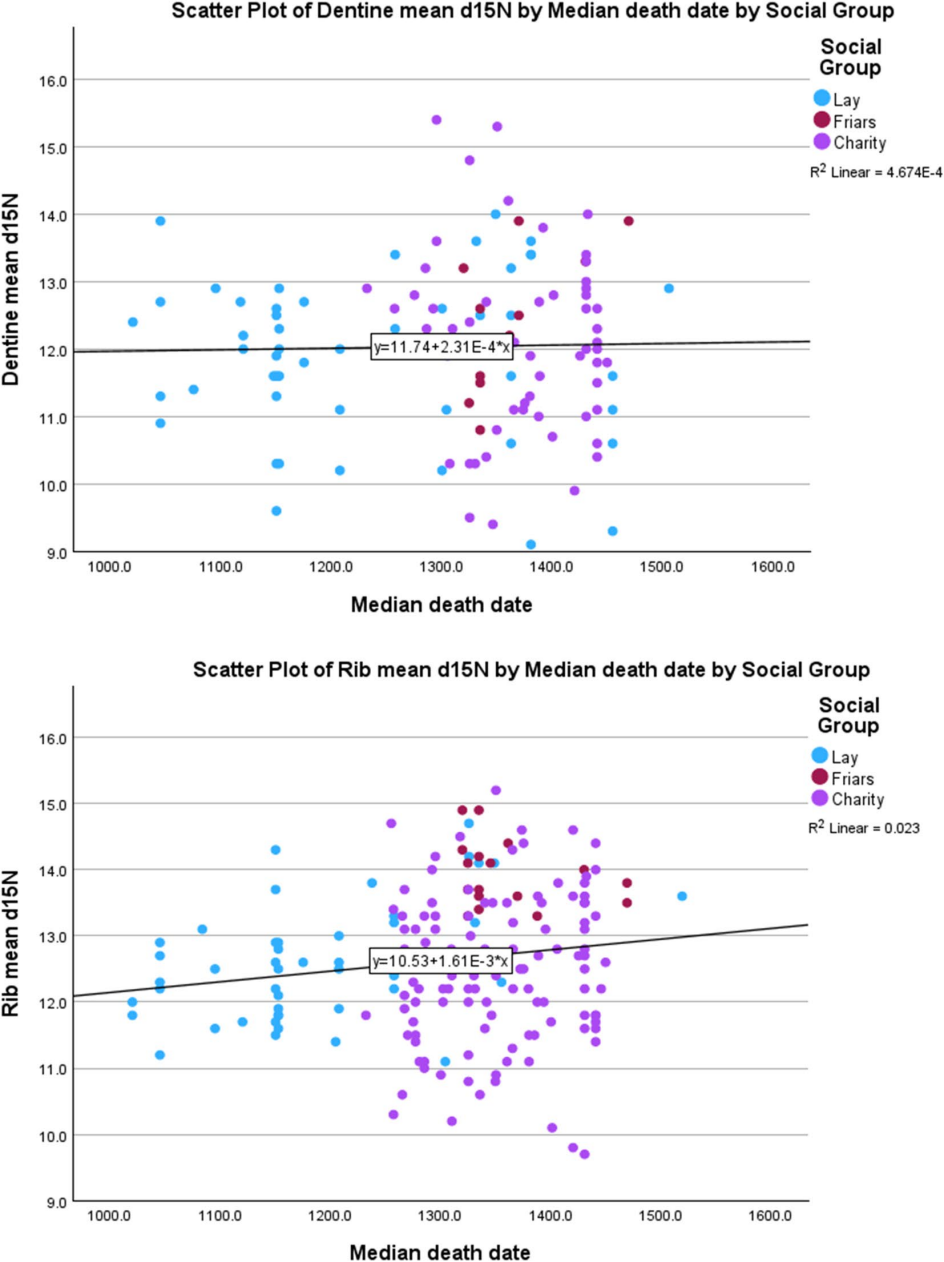

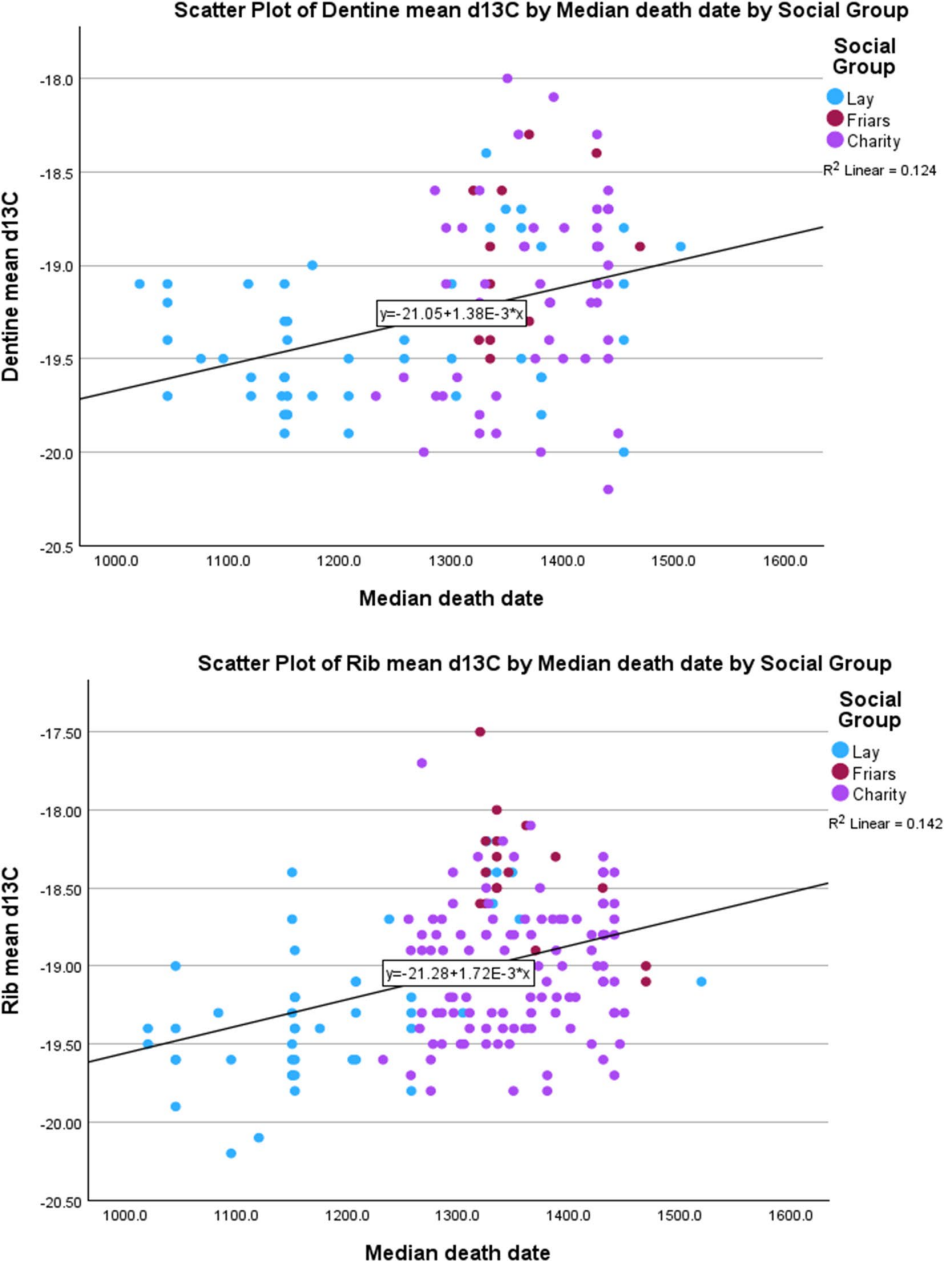
Isotopic data. (**a**) Dentine δ^15^N; (**b**) Rib δ^15^N. (**c**) Dentine δ^13^C. (**c**) Rib δ^13^C.

**Fig. 2:**
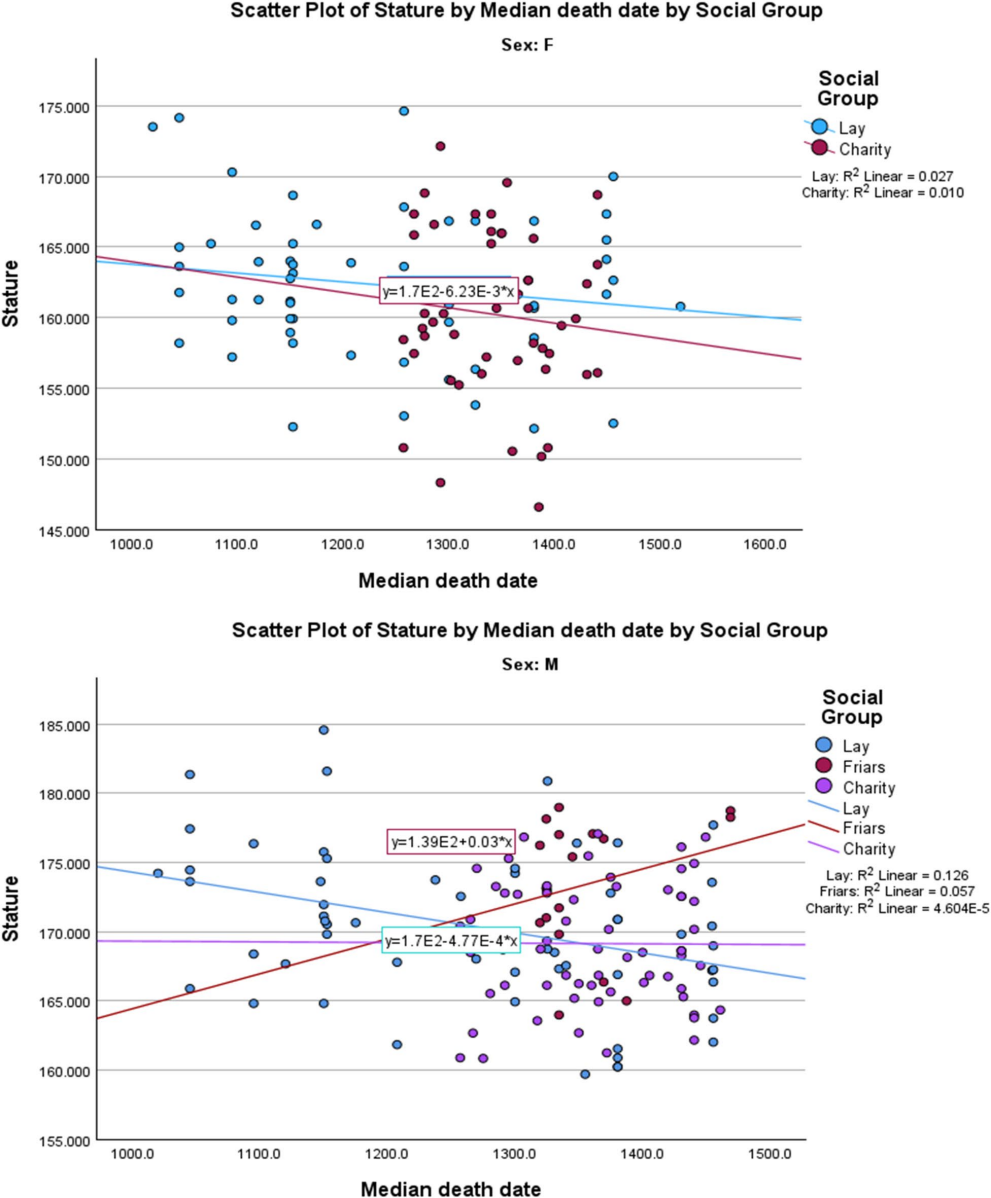
(**a**) Female stature. (**b**) Male stature.

Many basic bioarchaeological indicators also do not display significant changes (Figs. 1 and 2; Table 3, SI 4). Enamel hypoplasia is a defect of tooth formation indicating growth disturbance in childhood; it is often interpreted bioarchaeologically as a measure of generalised stress. The same is true of cribra orbitalia, a cranial lesion possibly indicating anemia, rickets or scurvy (among other causes). Maxillary sinusitis reflects chronic infection of the sinuses or inflammation from poor-quality air, perhaps from particulates in poorly ventilated housing. Periosteal new bone formation evidences a generalised reaction to infectious disease, localised injury, lung disease or other inflammatory conditions. All of these common bioarchaeological indicators of health show no significant change following the Black Death, nor after 1250 or 1300. The same is also true of tuberculosis—an important finding for public health, as this bacterial disease was a major cause of disability and death in medieval England, and was endemic in medieval Cambridge^52^.

**Table 3:** Before/ after comparisons (for summary, only p-values are given here; see supplementary information 5 for full data and statistical details).

Summary table	*p*-values for comparisons		
	Before/after 1250	Before/after 1300	Before/after plague
Enamel hypoplasia	0.753	0.412	0.571
Cribra orbitalia	0.428	0.704	0.623
Vitamin D deficiency	0.109	0.218	**0.035**
Maxillary sinusitis	0.14	0.524	0.697
Periosteal New Bone Growth	0.73	0.87	0.543
Tuberculosis/ respiratory infection	0.823	0.589	**0.064**
Tuberculosis	0.744	0.404	0.741
DISH	**0.059**	0.102	0.336
Hallux valgus	0.115	**0.09**	0.188
Extraspinal osteoarthritis	**0.006**	**0.01**	**0.048**
Trauma	**0.017**	**0.026**	0.123
Cranial trauma	**0.04**	**0.005**	**0.047**
Schmorl’s nodes	**0.035**	**0.008**	**0.02**
Adult age at death	0.353	0.243	0.89
Stature (female)	**0.08**	**0.03**	0.309
Stature (male)	**0.027**	**0.043**	0.181
D13C dentine	**0.001**	**0.001**	**0.011**
D13C ribs	**0.001**	**0.001**	0.246
D15N dentine	0.411	0.436	0.983
D15N ribs	**0.038**	**0.034**	0.312

Several apparent shifts may actually reflect changes in the balance of social groups in the sample. It is well-known that populations dying younger may actually display lower prevalences of age-related illnesses and of lesions which accumulated in the skeleton throughout the lifespan^53^. We take account of this factor in two ways. First, the “pre-plague” and “post-plague” samples do not show significant differences in age at death (see below). Secondly, we control for the effect of a changing mixture of sub-samples which may have different ages at death. The post-plague sample is characterised by greater representation of recipients of charity (Hospital residents); since these people died slightly younger, they have a lower rate of osteoarthritis than the general population. Consequently, when the prevalence of osteoarthritis declines in the later 14th -15th centuries, logistic regression (Table 4) suggests that the major factor influencing apparent temporal change is actually which social group individuals in the sample belong to. The same is true for the increased level of respiratory infections, which are more common in Hospital residents than in other groups. Traumatic injury presents a complex picture. It was more prevalent among males than females in all periods and groups; it was common among both laypeople and friars, reflecting the general hazards of living and working in medieval Cambridge. Both trauma and cranial trauma appear to decrease consistently throughout the medieval period (Table 3). However, trauma was less common among recipients of charity (Hospital residents), reflecting both their sheltered, possibly inactive life and their shorter lifespan, which gave them fewer years to accumulate healed fractures in their skeletons^47^. The prevalence of trauma among recipients of charity remains stable before and after the Black Death; the available samples for laypeople are too sparse to give a reliable idea of its prevalence among post-plague laypeople (Table 5). Thus, a possible downward trend in traumatic injury may reflect changes in the nature of the sample. Adult age at death is another important parameter of population health. There are no significant differences in age-at-death profiles across any of the chronological contrasts (before/ after 1250, before/ after 1300, and before/ after the Black Death). A possible shift towards a slightly younger age at death is actually due to increasing representation of Hospital samples in later samples, as people from the Hospital had a slightly younger age at death. Thus, many bioarchaeological indicators, including important ones such as enamel hypoplasia, tuberculosis, and adult age at death, suggest continuity and stability rather than dramatic change in the health of medieval people.

**Table 4:** Multivariate analyses for all indicators. Independent variables: sex, social group, and before/ after 1250, 1300, and 1349.

Dependent variable	Notes	Test	Independent variables: sex, social group and date				Independent variables: sex, social group and date				Independent variables: sex, social group and date			
			Before/ after 1250				Before/ after 1300				Before/ after Black death			
			*r* squared	*p*, sex	*p*, social group	*p*, before/ after 1250	*r* squared	*p*, sex	*p*, social group	*p*, before/ after 1300	*r* squared	*p*, sex	*p*, social group	*p*, before/ after Black Death
Enamel hypoplasia	presence/ absence of > 1 lesion	logistic regression (two categories)	*0.073*	0.284	0.171	0.762	*0.008*	0.819	0.839	0.633	*0.066*	0.923	0.178	0.345
Cribra orbitalia	presence/ absence	ditto	*0.093*	0.695	**0.047**	0.222	*0.073*	0.293	0.238	0.874	*0.053*	0.594	0.284	0.685
Vitamin D deficiency	ditto	ditto	*0.169*	0.584	1	1	***0.239***	0.751	1	0.259	*0.194*	0.431	1	0.311
Maxillary sinusitis	ditto	ditto	*0.08*	0.642	0.581	0.374	*0.034*	0.562	0.954	0.435	*0.054*	0.504	0.592	0.142
Periosteal New Bone Growth	ditto	ditto	*0.166*	**0.005**	0.863	0.754	*0.138*	**0.018**	0.919	0.945	*0.135*	**0.007**	0.997	0.259
Tuberculosis/ respiratory infections	ditto	ditto	*0.169*	**0.045**	**0.009**	**0.066**	***0.218***	0.264	0.115	0.999	*0.136*	0.231	**0.044**	0.314
Tuberculosis	ditto	ditto	*0.12*	**0.004**	0.58	0.388	*0.103*	**0.017**	0.63	0.245	*0.089*	**0.009**	0.698	0.396
DISH	ditto	ditto	***0.237***	**0.015**	0.158	0.577	*0.169*	**0.097**	0.272	0.976	***0.293***	0.997	**0.084**	0.543
Hallux valgus	ditto	ditto	*0.105*	0.28	0.662	0.216	*0.119*	0.309	0.449	0.249	*0.144*	0.227	0.204	**0.05**
Extraspinal osteoarthritis	ditto	ditto	*0.083*	0.603	**0.086**	0.273	*0.111*	0.911	**0.035**	0.524	*0.093*	0.961	**0.007**	0.495
Trauma	ditto	ditto	*0.048*	0.322	0.832	**0.076**	*0.072*	**0.087**	0.8	**0.04**	*0.046*	**0.046**	0.672	0.143
Cranial trauma	ditto	ditto	*0.084*	0.783	0.688	0.237	***0.22***	0.255	0.351	**0.019**	*0.048*	0.651	0.792	0.156
Schmorl’s nodes (categorical)	none, 1–6, 7+	logistic regression (multiple categories)	*0.12*	0.676	0.165	0.545	*0.135*	0.487	0.717	0.559	*0.191*	0.487	**0.022**	**0.003**
Adult age at death	18–25, 26–45, 45-	ditto	*0.03*	0.612	0.665	0.569	*0.039*	0.517	0.872	0.334	*0.031*	0.543	0.357	0.849
Stature (female)	numerical	ANOVA	*0.087*		0.127	0.854	*0.161*		**0.039**	0.828	*0.13*		**0.005**	0.371
Stature (male)	numerical	ditto	*0.141*		**0.006**	**0.025**	*0.14*		**0.017**	**0.057**	*0.09*		**0.045**	0.416
D13C dentine	numerical	ditto	*0.119*	0.947	0.572	**0.078**	*0.192*	0.883	0.876	**0.029**	*0.144*	0.395	**0.06**	0.143
D13C ribs	numerical	ditto	***0.391***	**0.029**	**0.019**	0.576	***0.448***	**0.007**	0.224	**0.016**	***0.367***	**0.014**	0.1	0.306
D15N dentine	numerical	ditto	*0.029*	0.65	0.445	0.62	*0.031*	0.461	0.539	0.643	*0.106*	0.907	0.199	0.531
D15N ribs	numerical	ditto	***0.224***	0.604	**0.006**	0.116	***0.254***	**0.071**	0.164	0.479	***0.232***	0.409	**0.003**	0.149

**Table 5:** “Whole town” approach comparisons for before/ after 1349 (see supplementary information 6 for full details).

Observation		Simple mean			Whole town mean		
		Before	After	Change	Before	After	Change
Linear enamel hypoplasia (> 1 lesion)		0.48	0.69	0.21	0.51	0.55	0.04
Cribra orbitalia		0.39	0.48	0.09	0.39	0.25	− 0.14
Trauma		0.38	0.27	− 0.11	0.43	0.20	− 0.23
Cranial trauma		0.12	0.05	− 0.07	0.11	0.02	− 0.09
Schmorl’s nodes	None	0.59	0.32	− 0.27	0.64	0.03	− 0.62
	1 to 6	0.32	0.43	0.12	0.21	0.03	− 0.18
	7+	0.09	0.25	0.16	0.15	0.95	0.80
Adult age at death	18–25	0.18	0.18	0.00	0.10	0.13	0.03
	26–45	0.40	0.44	0.04	0.42	0.45	0.04
	46+	0.42	0.38	− 0.04	0.48	0.41	− 0.07
Stature	f	161.92	160.49	− 1.42	163.30	163.14	− 0.16
	m	171.23	169.76	− 1.47	171.85	169.93	− 1.92
D13C (dentine)		− 19.29	− 19.06	0.23	− 19.34	− 19.28	0.05
D13C (rib)		− 19.00	− 18.90	0.09	− 19.30	− 18.78	0.52
D15N (dentine)		12.07	12.04	− 0.04	12.00	11.94	− 0.06
D15N (rib)		12.72	12.77	0.05	12.66	13.81	1.15

Other changes may represent genuine historical developments, but ones principally of local significance. In these samples, Vitamin D deficiency is known entirely from a small number of cases found only among recipients of charity at the Hospital of St. John. Detailed bioarchaeological profiling of the Hospital’s residents reveals that the institution’s managers favoured specific categories of people considered particularly worthy of charity, including young, chronically ill people (especially females), working people who had fallen into poverty late in life, and, particularly in the later 1300s and 1400s, aged or ill university scholars^22^. Given that Vitamin D deficiency is not known in other groups, it seems likely that its occurrence late in the Hospital sequence represents the Hospital’s decision-makers profiling specific forms of poverty and illness rather than major developments in public health. The same is true for the rise in Schmorl’s nodes in the spine (an osteological consequence of herniated intervertebral disks) in the later 14th -15th century, which is localised primarily in a small group of older Hospital residents and may reflect an increasing emphasis on awarding charity to older working people.

Finally, some general changes in health are present, but they either are clearly traceable to specific causes, or they change at different times. DISH (Diffuse Idiopathic Skeletal Hyperostosis) is a mostly asymptomatic metabolic condition involving the ongoing formation of new bone, particularly in the vertebrae, which in fully-developed cases can partially fuse into a rigid bony column. In medieval and post-medieval collections generally, it is prevalent mostly in older males, and has been found to be commoner in later medieval samples, though the cause is unclear^54^; findings from Cambridge conform to this generalisation. Hallux valgus is a deformity of foot bones related to bunions; clinically, a major cause is wearing pointed footwear. In medieval Cambridge, hallux valgus increases throughout the time span examined; it is more common in the later 14th and 15th centuries. While aggregated data does not always show a clear increase, this is in part because it remains stable in females, and increases primarily in males. In medieval England, pointed footwear became increasingly fashionable in the 14th and 15th centuries, principally for men, and this underlies an increasing frequency of hallux valgus^44^.

Isotopic analysis of the skeletal material does not suggest major dietary change linked to events in the 14th century. Isotopic values in biological tissues provide a rudimentary indication of diet, with nitrogen isotope values in particular linked to animal protein consumption. These values reflect a broad average of the composition of food consumed, and major, sustained changes in dietary regime may be required to be visible isotopically; what may be considered a large change socially, such as an improvement in food quality, may not be visible. There is historical evidence that the consumption of meat and fish was socially significant in the medieval period, and previous work has established that different groups in medieval Cambridge^55,56^ and elsewhere^57^ are distinguishable isotopically. Here, however, when the data are considered by death date, we do not see major overall trends in the isotopic data. This is particularly clear for the dentine samples, which reflect childhood diet (Fig. 1a). The nitrogen isotope values measured in rib bone samples, which largely reflect adult diet, show a very slight trend upwards through time. However, these are biased by the later medieval Augustinian friars, who appeared to have shared a homogeneous, meat and/or dairy- and fish- rich diet (Fig. 1b. Once this social difference is taken into account, there appears to be no great change. Carbon isotope values measured in both rib bone and dentine samples show a slight increase over time (Fig. 1c and d). This appears to be driven at least in part by the higher carbon isotope values exhibited by the Augustinian friars due to the inclusion of marine proteins in their diet. While this may perhaps suggest that the overall basis of medieval diet did not change greatly, the most obvious pattern—which should not be biased by such interpretive debates -- is not the small increase in average values, but the overall greater *range* in both carbon and nitrogen isotope values through the sequence. This may reveal an expansion in the range of dietary habits resulting in greater differences among individuals, perhaps related to greater social differentiation in food consumption, particularly marine fish^58^. However, this begins before the Black Death (Fig. 1a–d; Tables 3 and 4) and it is likely to be more closely linked to religious rules around certain food items for certain groups, as well as changes in food fashions and availability of foodstuffs.

The most unexpected historical trend concerns stature. Adult stature is a sensitive indicator of childhood health, nutrition and environment, particularly within genetically homogeneous groups such as the medieval population of Cambridge. Research has already shown that social groups within medieval Cambridge differed in stature, with religious professionals about 2 cm taller than lay males and both lay males and lay females about 2 cm taller than recipients of charity^21^. Chronologically, both male and female stature decline through the period studied. The decline is statistically meaningful in comparisons of before and after 1250 and 1300; it appears to have stabilised around 1350, when before/ after differences are minimal once the balance of social groups in the sample is adjusted for (cf. the “whole town” mean, Table 5 and multivariate analyses, Table 4). Regression analysis in fact shows the decline in stature clearest in the “laypeople” subgroup (Fig. 2). This suggests a major decline in health and well-being in the period preceding the Black Death—the late 1200s and early 1300s.

## Discussion

Imagine that you set up a table in Cambridge’s marketplace on a busy day in 1300 and ask several hundred people to complete a broad health questionnaire. You then repeat the exercise in 1400. What would this reveal about the long-term consequences of the catastrophic 1348-9 Black Death epidemic?

What we have tried to do here is the skeletal and molecular equivalent of such an exercise. We anticipated three possible outcomes: continuity, dramatic change driven by the Black Death, or some more complex mosaic of change and continuity. Finding out which the data reveal involved complex intermediary steps. As much as opinion pollsters, bioarchaeologists are subject to the vagaries of which subjects answer their questionnaire; in a heterogeneous social landscape such as medieval England, the available dataset almost never represents the population in a simple or static way. Combining multiple statistical techniques allowed disentanglement of historical change from sample biases. Moreover, it is important not to assume that all 14th -century change must be due to the Black Death. The most direct screening tool is simply to verify that a potential change does or does not in fact coincide chronologically with the epidemic.

This research revealed two important findings. First, many aspects of life do not display significant change (Table 6). This is true of minor, chronic health problems (e.g. maxillary sinusitis, extraspinal osteoarthritis), skeletal features which can indicate minor or major problems (e.g. enamel hypoplasia, cribra orbitalia, periosteal new bone formation, respiratory infections) and indicators of major health problems (e.g. tuberculosis, traumatic injury). Adult age at death appears not to have risen or fallen significantly. While it is always possible that larger samples might reveal other trends, the samples analysed here suggest that the basic framework of life appears not to have changed dramatically. This may not be surprising, given that medieval life for many ordinary townsfolk entailed strenuous physical labour, crowded housing, an environment awash in pathogens, and the possibility of hunger.

**Table 6:** Summary table of results and interpretations (bioarchaeological indicators which showed significant historical change are highlighted in bold).

Skeletal indicator	Results, possible causes and notes
Population genetics	No chronological trend^51^
Enamel hypoplasia	No chronological trend
Cribra orbitalia	No chronological trend; more prevalent in people receiving charity
**Vitamin D deficiency**	**Increases in sample in later 14th -15th century, possibly due to changes in how recipients of charity were selected**
Maxillary sinusitis	No chronological trend
Periosteal new bone growth	No chronological trend; may be more prevalent in women recipients of charity
Respiratory infection	Apparent increased frequency post-1349 probably due to changing balance of social groups in sample, particularly increased inclusion of female recipients of charity
Tuberculosis	No chronological trend; more prevalent in women^52^
**DISH**	**More prevalent after 1250 than previously; no clear 14th century change. More prevalent in males throughout, and late in sequence possibly more common in Hospital males**
**Hallux valgus**	**More prevalent in 14th -15th century, especially in males, related to lifestyle changes (fashionable footwear)** ^44^
Extraspinal osteoarthritis	Apparent increased frequency post-1349 probably due to changing nature of sample: increasing proportion of recipients of charity
**Trauma**	**More prevalent in males throughout; less common in Hospital residents** ^59^
**Cranial trauma**	**More prevalent in males throughout; less common in Hospital residents** ^59^
**Schmorl’s nodes**	**Changes in later 14th -15th century are found predominantly in recipients of charity, possibly due to changes in how recipients of charity were selected**
Adult age at death	No chronological trend
**Stature**	**Both female and male stature decrease throughout period, beginning before 14th century.**
Nitrogen isotope values	No chronological trend; apparent increase reflects inclusion in later sample of religious professionals (Augustinian friars) with higher d15N values.
**Carbon isotope values**	**Increased range of consumption practices in social landscape of later medieval Cambridge (from around 1300 onwards), as well as inclusion in later sample of religious professionals (Augustinian friars) with higher δ13C values**

Continuity also appears in major aspects of social life and diet. The genetic composition of Cambridge shows no change, suggesting that there was no significant exogeneous population turnover and that, severe though it was, the plague epidemic did not exert a significant selective effect^51^. Diet is particularly interesting. Meat was a recognised element in the quality of medieval life, with higher-status people consuming more (a difference attested isotopically in archaeological material from medieval Cambridge^53^. Indeed, when labour became scarce after the Black Death, commentators observed, usually in scandalised tones, that harvest workers now expected meat as well as bread, cheese and ale in the meals their employers traditionally provided for them^11^. Such anecdotal observations have been taken as evidence for the improved status of labourers, but it is hard to know whether they actually attest a general, widespread dietary upgrade. Lack of significant change in nitrogen isotope values may suggest that diet did not change sharply. However, it is possible instead that people were choosing choicer cuts of meat; such improvements would be isotopically invisible.

For evidenced biosocial change in the later 14th -15th centuries, the pattern is of complex, often quite local social histories rather than sweeping social transformation. Some changes may reflect the increasing size, complexity and prosperity of Cambridge, particularly in the 14th and 15th centuries. The fast-growing university and other religious foundations brought money and demand for skills, goods (including high-end foods) and services into the town. Thus, changes in the range of carbon isotope values suggest an increased scale of social differentiation in food consumption habits; this trend clearly began before the Black Death epidemic. Among changes which post-date the Black Death, changes in Vitamin D deficiency and Schmorl’s nodes are specific to particular sectors of the population, specifically residents of the Hospital of St. John; this suggests that they are more parsimoniously explained through changes in how recipients of charity were selected than to general, widespread trends in population health. Late medieval increases in hallux valgus, specifically in males, are plausibly related to the fashion for pointed-toed footwear for men at this time. Such changes call into question the logic of assuming that changes following the Black Death must be consequences of the Black Death. With the eye of faith, one could potentially relate shifting theological theories of charity to the Black Death and new clothing fashions to increased disposable income, but the evidential linkages are rather attenuated. At the very least, if these are the principal skeletal affects attributable to the Black Death, it is hardly a strong argument for sweeping biosocial transformation.

The second major finding was more unexpected. Adult stature declined, particularly for males, and it clearly began to decline well before the Black Death. Both female and male stature were about 3 cm greater before 1250 CE than after that date. Different subgroups within the community differed in stature, but the “whole town” mean shows reliably that by about 1350, women’s stature had stabilised around 163 cm, while males was still falling (171.85 cm before 1350, 169.93 cm after 1350). The context for this downward trend is clear. There is broad consensus that by sometime in the 13th century, high population levels in England had pushed much of the population to the brink of precarity, with farmers working increasingly small holdings and every poor harvest a potential episode of hunger^12,14,60^. Thus, declining stature from the 13th century onwards may reflect deteriorating living conditions. In this context, one thing which remained constant throughout the study period was poor health. However one subdivides the sample chronologically and socially, at least half the people observed had experienced more than one childhood episode of hunger or illness serious enough to interrupt their tooth formation, and about two in five had experienced childhood anemia, illness or metabolic disturbance serious enough to cause porosity in the orbits. About one in ten evince skeletal evidence of tuberculosis; since skeletal signs manifest in only a small fraction of people suffering from tuberculosis^37^this implies that a sizeable portion of the population suffered from this serious disease. Both before and after the Black Death, the bioarchaeological data portray a population under stress from disease and possibly hunger. The stature data thus confirm that change independent of the Black Death was happening, in this case an ongoing, gradual worsening of living conditions.

Such data correspond well with Campbell’s “perfect storm” model of a general 14th century crisis^12^. In this model, by the 1200s, Western Europe had reached the limit of population possible with food production methods of the time. In such marginal conditions, even a small downturn was enough to cause higher food prices and famine. The climatic deterioration attested in tree-ring data and Arctic ice core isotopes led to a series of poor harvests resulting in recurrent famines and leading to a weakened, vulnerable population. Another consequence was the emergence of new pathogens, both human and animal, including *Yersinia pestis*, whose transmission ecology in Central Asia had been disturbed by climate change^61^. This model does not deny that the Black Death had major consequences, particularly demographic ones, which affected European society for several centuries afterwards. But it places the plague within a larger context, showing that its effects may actually have depended in part on long-term biosocial processes already going on for a century or more.

Bioarchaeological data emerging from several studies broadly confirm such a model. Groups in London^14,17,19,20^ may also evince declining health before the Black Death. Within such a model, the Cambridge data show the complexity of historical change. Responses following the plague seem more varied. In the face of catastrophe, people carried on with familiar ways of life. Global rebalancing of population and land may have taken half a century to escape short-term, local fluctuations and reach a stable, widespread new equilibrium^6^. In the meantime, major causes were mediated through local social structures, affecting different groups differentially. Elsewhere, health may have improved following the epidemic^16,17,19^. In Cambridge, little change is evident. After the plague, some groups may have spent disposable income on fashion or upgraded their food consumption; increased economic opportunity in a urban landscape may be visible not as across-the-board improvement but as increased variation between groups. Thus, just as the impacts of historical change varied among sectors and subgroups, they may have varied regionally. Cambridge represents a town rather than the rural contexts in which 80–90% of medieval people lived, and a town with an unusual economic basis which may have prospered while others stagnated.

We would never minimise the human cost of the Black Death in suffering and grief, and many of its demographic, social and economic changes are well-documented historically. But the traumatic loss epidemics cause may not be proportional to their real long-term influence on human lifestyle. Skeletal remains from medieval Cambridge and its hinterland show no major changes which can be attributed directly to the Black Death. Instead, they attest major continuity of lifeways, confirming the general picture of continuity supplied by archaeologies and histories of daily life. They also show the effects of other, more local trends in social history, and, importantly, of long-term changes in human health preceding, and potentially helping to cause, the disastrous epidemic.

## Methods

### Archaeological materials

Skeletal series studied here included people from varied contexts in medieval Cambridge. Because representation of non-adults in archaeological burials varied widely between sites studied, only adults were included in this study. This series represents a large social cross-section of medieval people. It is subject to limitations which affect the field generally. While most people in medieval England lived rurally, most skeletal assemblages available for analysis represent urban contexts, as do ours here. For similar preservational reasons, more samples are available to represent the 11th -14th centuries than to represent the 15th -17th centuries. Moreover, archaeological dating of burials is necessarily approximate. In this study, each skeleton was dated to as narrow an interval as possible using archaeological evidence, using the site stratigraphy and overall chronology anchored in radiocarbon dates. It was then categorised as dating to before or after various historical break points (1250, 1300, 1350, 1400). The “before” or “after” categorisations used in statistical analysis, thus, represent a categorical assessment grounded in archaeological data; burials spanning the break points for each comparison were excluded from statistical tests of change at that point. For certain analyses, intervals were converted to point estimate dates (see “Statistical methods” below). When point estimates of a burial’s date were needed, the median of the burial’s possible timespan was used. This provided data which could be scatterplotted. However, such point estimates tend to bunch dates artificially towards the middle of their date range, and statistical testing thus relied on archaeologically grounded chronological categories rather than point estimates of dates.

One additional source of bias affecting human remains comes from the fact that the different tissues in the human body date to different points in the lifespan. For example, if a person was born in 1320 and died aged 60 in 1380, their dental isotopes and adult stature would reflect their pre-Black Death childhood environment of the 1320–1330s, while their bone isotopes and age at death would reflect their post-Black Death adult world of the 1360–1370s and their skeletal pathologies and traumas could reflect the entire interval in between. However, this is likely to bias data for only a limited number of long-lived 14th -century people, and given the margin of error involved with both archaeological dating and skeletal age determination, it is preferable to tolerate it as a known issue than to “correct” the data ad hoc for such cases.

Overall, 330 adults from between 1000 and 1500 CE were analysed (see Supplementary Information Table S1). Individuals were grouped into three social categories (ordinary people, recipients of charity, and religious professionals), though these categories clearly overlapped in life as people moved in and out of them.

In our sample of 330 people from between 1000 and 1500 CE, ordinary people are represented by people buried at a local parish church, All Saints By The Castle (1000–1365, *n* = 91)^62^. This was a church cemetery containing parishioners from an outlying, semi-rural neighbourhood of Cambridge north of the river Cam. The cemetery came into use around 1000 CE and was used until 1365-6, when the parish, severely reduced in population by the Black Death (1348-9) and the subsequent epidemic in 1362, was merged with a nearby one. At least one individual studied from All Saints died in the Black Death epidemic^62^but the great majority pre-date the epidemic. A few additional samples representing townsfolk came from the Augustinian Friary^63,64^ (1280s-1538, *n* = 12) and from Hostel Yard (*n* = 4). The former were relatively prosperous townsfolk who had requested burial at the city’s Augustinian Friary rather than at their parish church, normally making a special donation to the Friary for the privilege. The latter were buried in the cemetery of St. Bene’t’s Church in circumstances suggesting that they comprised a small mass burial associated with the Black Death^65^; aDNA of *Yersinia pestis* was found in their skeletons. In this study, their skeletons were considered “pre-plague”, as they reflect conditions of growth and life experienced before the epidemic. Finally, 50 individuals from the rural village of Clopton^66^approximately 20 km southwest of Cambridge, were included to represent people with a more rural lifestyle; some residents of Cambridge would also have worked in agriculture and there was considerable mobility between town and country.

Religious professionals include friars from the Augustinian Friary (1280s-1538, *n* = 18), who were identified positively as friars by the garments in which they were buried^63,64^.

Poor people are represented by 155 burials from the Hospital of St. John (1190–1200 to 1511). The Hospital was a charitable institution which provided shelter, food and clothing for selected people who were poor and/or ill, and deemed worthy of charity^67,68^. Its burial population was highly varied, including some very poor people, some who lived much like other working folk in Cambridge until they were overtaken by adverse circumstances, and some old or ill university scholars^22,69,70^ Given that medieval Cambridge must have contained many people in need, one key question is how specific individuals were chosen to reside there; there is some evidence that the criteria used to profile recipients of charity may have changed throughout the medieval period. A few people who were not resident in the Hospital were also buried there.

These three groups—ordinary people, religious professionals, and people living on charity -- differed significantly in their health and lifestyle^21^. Compared to ordinary townspeople, the poor living within a charitable institution were significantly shorter, had more indications of childhood poverty, and often died younger; they had fewer diseases of age and activity-related accidents. The friars were members of a wealthy institution since adolescence; however, members of the Augustinian order also worked at manual occupations. They shared the townspeople’s diseases, manual labour, high accident rates and mixed ages at death, but were significantly larger and better nourished.

In assessing historical change, such differences are important. While the Hospital residents make up many of our samples, they would have been a small minority of the city’s population; the converse is true of the townspeople. Moreover, the proportions of each group making up the archaeological sample available for analysis change throughout the medieval period, so that apparent historical changes must be calibrated against the composition of the sample. These issues are addressed statistically below.

### Bioarchaeological methods

A range of standard bioarchaeological indicators of health, activity and environment were used (Table 4). In general, levels of skeletal preservation were good to excellent in all skeletal samples analysed, and there were no substantial disparities of preservation between sites.

As Wood et al.^53^ point out, such indicators intersect with a sample’s demographic characteristics in complex ways (for instance, longer-lived samples often display higher prevalences of some palaeopathologies representing slow, chronic conditions, age-related conditions, or lesions which accumulate in the skeleton over time), and higher prevalences of non-lethal conditions may actually indicate a population of hardy survivors. Here, as noted in the text, we establish that the samples compared do not have significant demographic differences; when sub-groups might, we use multivariate analysis to take this into account. Comparing temporal periods also implicitly assumes that the demographic structure of the population is stationary, without major changes in age structure or composition due to incoming or exiting population. Such an assumption is necessarily an approximation in all bioarchaeological interpretation. Here, population is demonstrably non-stationary, since it underwent a significant crash in 1348-9. However, this should not bias the comparisons involved unless the populations before and after differed in important ways. For example, if there was increased population movement between rural areas and towns after 1348-9, this would bias the results only if the groups involved differed in age structure and/or health; both here and for medieval England in general, materials are lacking to prove or disprove this possibility, and so we can merely mention it at present.

Methods of isotopic study are fully outlined in previous publications^52,53^. In brief, bulk samples of rib bone and dentine from either second premolars or second molars were selected from individuals from the target populations. Sample preparation and collagen extraction was carried out following McDonald Institute protocols, based on a modified version of the Longin method^71–73^. Measurement was carried out using a Costech automated elemental analyser coupled in continuous-flow mode with a Thermo Finnigan MAT253 isotope ratio mass spectrometer. All samples are reported on the international scale relative to VPDB for carbon and AIR for nitrogen^74^. Based on replicate analyses of standards, analytical error was < ± 0.2‰. All results were compared to established quality indicators to see if they were within the acceptable atomic C: N ratio range of 2.9-3.6^75^ and above the minimum acceptable %C and %N threshold (taken as > 13% for C and > 4–5% for N^76^.

The samples analysed here have also been the subject of genetic study specifically examining historical change in the medieval period^51^; methods of genetic analysis and the resulting data are fully explained therein and results are only referred to here.

A substantial number of samples were analysed for strontium isotopes as a source of information upon human mobility^56^. These successfully identified individuals of “non-local” origin, in some cases from long distances away^22,56^. However, we do not consider strontium isotopic data further here. While long-distance “non-local” individuals are observable throughout Cambridge’s medieval history, their number is too low to permit statistical analysis of change. Moreover, most of the post-plague mobility hypothesized by historians would be relatively short-distance (for instance, people moving to towns within 40–50 km of their natal villages). East Anglia is homogeneous geologically and all the areas involved in such mobility would share similar geochemical signatures, making mobility over such distances from Cambridge undetectable using strontium or oxygen isotopic analysis.

### Statistical methods for data analysis

Like all archaeological data, the data analysed here present statistical challenges. Each datapoint, representing one individual, includes multiple observations tied to a chronological interval representing their possible burial dates.

Every approach to analysing change in such data has advantages and disadvantages. For example, categorising each burial archaeologically as “before” or “after” a chronological cut-point works with the most solid chronological evidence available for each individual, but it focuses upon the date when they died and were buried rather than when they were born, grew and lived. It allows straightforward comparison using standard statistical methods for categorical data, but it assumes that samples remain comparable between periods in all other ways, it presumes discontinuous or blocky patterns of change, it hinges upon choosing the right cut-point, and it loses datapoints which straddle the cut-point—potentially a serious loss of data. Conversely, converting a burial’s possible date range to an estimated point estimate makes it possible to visualise continuous trends, but it implies spurious precision and it can distort chronological patterns by bunching burials towards the centre of their range.

As a further complicating factor, archaeological samples almost never represent a long timespan without some change in their social composition, confounding simple comparison between chronological periods. Here, most samples for the period 1000–1300 are ordinary people from a parish church cemetery, All Saints; most samples for the period 1300–1500 are recipients of charity from the Hospital of St John, a charitable institution. Since the two sites partly represent different kinds of people, do changes over the medieval period reflect real historic change in how people lived or the changing balance of archaeological samples available to us?

The approach adopted here is to use multiple techniques of data exploration, seeking robust results. Four approaches were used.

*1. Outlining trends visually and statistically*,* using point estimates of burial date.* To create point estimates for a burial’s date, the simplest approach is to use the midpoint (median) of its possible date range. While such point estimates should be treated only as approximate, they enable analyses and visualisations:

- For categorical variables such as presence/ absence of a skeletal trait: histograms and boxplots showing the distribution over time of people with and without the trait (Supplementary Information 4).
- For numerical variables such as stature and isotopic ratio values, scatterplots of the values vs. date, with relevant trend lines. (Figs. 1 and 2)

Such comparisons can also be tested for patterning, for instance by calculating correlation coefficients between numerical values and dates (Fig. 1).

2. *Before/ after comparisons of categorised data.* Each burial was categorised chronologically; for example, burials were classified as occurring before or after the 1348-9 epidemic. Burials which straddled a given cut-point evenly were excluded from that comparison. The grouped burials before and after the date were then compared, and the probability of change for each form of data at each cut-point was evaluated using standard tests for (χ^2^ for nominal data, Student’s T for numerical data). Following American Statistical Association recommendations^77^we do not engage in formal hypothesis testing; p-values are simply used as a gauge of relative probability.

To avoid assuming teleologically that change must have happened only before and after the Black Death epidemic in 1348-9, data were also categorised as before and after other cut-points (1250 and 1300), and all bioarchaeological data were compared using these cut points (Table 3, Supplementary Information 5). (Too few early and late samples were available to make before/ after comparisons possible for 1200 and 1400).

3. *Using the “whole city” approach.* Archaeological samples rarely represent a community’s population proportionally. Moreover, simply calculating prevalences using all available data risks confounding chronological change with change in the nature of the available samples. As noted above, the proportion of ordinary people vs. recipients of charity in the available sample changes throughout the timespan examined, and the two groups have different health profiles for some bioarchaeological variables^21^ (Supplementary Information 3). The sex ratio of the sample also varies over time, and females and males differed in some health characteristics (Supplementary Information 2). A simple corrective is the “whole town” approach, which combines different subgroups proportionately to construct a weighted average^16^. Data for each group are weighted according to how much each group is estimated historically to have contributed to the overall population of the city: townsfolk (estimated as 85% for males, 99% for females), recipients of charity (1% for males, 1% for females), and religious professionals (14% for males, negligible for females). As detailed census information is not available for the medieval period, these estimates were arrived at simply by considering the known elements of the city in very approximate terms. In most medieval towns, ordinary clergy may have made up perhaps 1–2% of the adult male population. Cambridge had at least 12 parish churches; 15 active and old parish priests and associated staff is reasonable. Cambridge also had sizeable religious houses from almost all major religious orders (Benedictines, Carmelites, Domenicans, Augustinians, Franciscans, and others), totalling perhaps 100–200 male clerics (with one relatively small nunnery, the Benedictine St. Radegund’s, the comparable figure for women is statistically negligible). A third form of male religious professional was University scholars. The University totalled between 300 and 700 in the timespan considered here, though a sizeable proportion would have been students rather than long-term, employed teachers. A conservative overall estimate might be between 200 and 400 male clerics in a town of 1000 to 2000 adult males (3000–5000 total people). All of these figures are approximate and all would have varied over time, but an overall estimate of between 10 and 20% of adult males as professional clerics is reasonable; we have chosen an estimate on the conservative side here. Similarly, Cambridge had between one and three operational hospitals at a given moment in the medieval period, each of which probably held 8–12 residents, so an estimate of perhaps 1% of the town’s population living on charity is reasonable. Results were calculated separately for males and females and then averaged to make a mid-sex mean representing the whole community. The resulting “whole town” averages give a balanced picture of the overall health of the group before and after the plague.

For some indicators, data were too sparse to subdivide the available samples further into subgroups, but the “whole town” approach gave useful results for some of the most important indicators, particularly stature and isotopic data. Results are presented in Table 5 and Supplementary Information 6.

4. *Multivariate methods for testing historical change.* To help unravel the nexus of related factors, we used multivariate methods to assess the importance of sex (males vs. females), social background (ordinary people, recipients of charity, religious professionals) and time (before/after a given date). These three categorical forms of data were used as independent variables for logistic regression (for nominal dependent variables) and for ANOVA (for numerical dependent variables). The resulting correlation coefficients and associated p-values give a sense of whether the three factors cumulatively account for differences in the dependent variable and of how important chronological period was compared to other possible causes for change. Tests were run using 1250, 1300 and 1350 as potential inflection points for change. Results are presented in Table 4.

*Overall logic of interpretation*. Disentangling skeletal data can be complicated. Here, once a possible historic change is identified, attention is turned to two related questions:

- Identifying its chronological pattern. Given the wide chronological range of many archaeological burials, the fact that data within the skeleton may refer to different periods in the subject’s lifespan, and the imprecision of skeletal age determination, it may be difficult to identify a point at which a historical trend began. However, if the pattern of change is more marked in comparisons of data before/ after Date X than it is in comparisons of data before/ after Date Y, it makes sense to conclude that Date X represents a better point of inflection for the change. Similarly, if logistic regression shows a more significant factor weighting for before/ after contrasts for Date X than for Date Y, it suggests that, all else being equal, whether a burial occurred before or after Date X exerted a stronger influence on the outcome than whether it occurred before or after Date Y.
- Identifying whether change is general or localised within a sector of Cambridge’s heterogeneous population. This is relevant both methodologically, as representation of different groups within the study sample changes over time, and substantively, as it is assumed that important social effects are likely to affect more than one subgroup. This can be assessed in two ways, besides simply inspecting statistics for each sub-group. First, if the “whole town” weighted average representing different population sectors proportionately differs much from the simple overall average, it is likely that the change is localised within one sub-sector which is over- or under-represented in the overall average. Secondly, in logistic regression and ANOVA, the factor significances for “social group” can be compared with significances for other factors such as date.

## Supplementary Information

Below is the link to the electronic supplementary material.

Supplementary Material 1

Supplementary Material 2

## Data Availability

The datasets used and/or analysed during the current study available from the corresponding author on reasonable request.
