# Supplementary material for: More continuity than change following the Black Death epidemic in medieval Cambridge
Source: Sci Rep. 2025 Sep 29;15:33388. doi: 10.1038/s41598-025-18437-5 (PMC12480250; doi:10.1038/s41598-025-18437-5)
Supplement: Supplementary file 1 — Supplementary Material 1 [file 41598_2025_18437_MOESM1_ESM.docx]

**More continuity than change following the Black Death epidemic in medieval Cambridge**

John Robb, Jenna M. Dittmar, Sarah A. Inskip, Alice K. Rose, Piers D. Mitchell, Tamsin C. O’Connell, Mary Price, Craig Cessford

**Supplementary Information**

Supplementary Information 1. Description of the dataset

Supplementary Information 2. Distribution of all indicators by sex

Supplementary Information 3. Distribution of all indicators by social group

Supplementary Information 4. Graphic representations of all data

Supplementary Information 5. Data and statistical details for before/ after comparisons reported in Table 2

Supplementary Information 6. Data and statistical details for “Whole town” comparisons for before/ after the Black Death reported in Table 3

**Supplementary Information 1. Descriptive summaries of the dataset**

| Site | | | Social Group | | | Total |
| --- | --- | --- | --- | --- | --- | --- |
|  |  |  | Charity | Friars | Lay |  |
| All Saints | Sex | F |  |  | 48 | 48 |
|  |  | M |  |  | 43 | 43 |
|  | Total | |  |  | 91 | 91 |
| Clopton | Sex | F |  |  | 18 | 18 |
|  |  | M |  |  | 32 | 32 |
|  | Total | |  |  | 50 | 50 |
| Hostel Yard | Sex | F |  |  | 1 | 1 |
|  |  | M |  |  | 3 | 3 |
|  | Total | |  |  | 4 | 4 |
| New Museums | Sex | F |  | 0 | 5 | 5 |
|  |  | M |  | 18 | 7 | 25 |
|  | Total | |  | 18 | 12 | 30 |
| St Johns Hospital | Sex | F | 63 |  |  | 63 |
|  |  | M | 92 |  |  | 92 |
|  | Total | | 155 |  |  | 155 |
| Total | Sex | F | 63 | 0 | 72 | 135 |
|  |  | M | 92 | 18 | 85 | 195 |
|  | Total | | 155 | 18 | 157 | 330 |

| Pre/post plague | | | Social Group | | | Total |
| --- | --- | --- | --- | --- | --- | --- |
|  |  |  | Charity | Friars | Lay |  |
| ante | Sex | F | 34 | 0 | 30 | 64 |
|  |  | M | 37 | 12 | 37 | 86 |
|  | Total | | 71 | 12 | 67 | 150 |
| post | Sex | F | 16 | 0 | 12 | 28 |
|  |  | M | 33 | 6 | 16 | 55 |
|  | Total | | 49 | 6 | 28 | 83 |
| Total | Sex | F | 50 | 0 | 42 | 92 |
|  |  | M | 70 | 18 | 53 | 141 |
|  | Total | | 120 | 18 | 95 | 233 |

**Supplementary Information 2. Distribution of all indicators by sex**

|  | | | More than one hypoplastic lesion | | Total |
| --- | --- | --- | --- | --- | --- |
|  |  |  | .00 | 1.00 |  |
| Sex | F | Count | 18 | 31 | 49 |
|  |  | % within Sex | 36.7% | 63.3% | 100.0% |
|  | M | Count | 39 | 50 | 89 |
|  |  | % within Sex | 43.8% | 56.2% | 100.0% |
| Total | | Count | 57 | 81 | 138 |
|  |  | % within Sex | 41.3% | 58.7% | 100.0% |

|  | | | Cribra orbitalia | | Total |
| --- | --- | --- | --- | --- | --- |
|  |  |  | 0 | 1 |  |
| Sex | F | Count | 29 | 28 | 57 |
|  |  | % within Sex | 50.9% | 49.1% | 100.0% |
|  | M | Count | 75 | 43 | 118 |
|  |  | % within Sex | 63.6% | 36.4% | 100.0% |
| Total | | Count | 104 | 71 | 175 |
|  |  | % within Sex | 59.4% | 40.6% | 100.0% |

|  | | | Vitamin D deficiency | | Total |
| --- | --- | --- | --- | --- | --- |
|  |  |  | 0 | 1 |  |
| Sex | F | Count | 93 | 4 | 97 |
|  |  | % within Sex | 95.9% | 4.1% | 100.0% |
|  | M | Count | 138 | 7 | 145 |
|  |  | % within Sex | 95.2% | 4.8% | 100.0% |
| Total | | Count | 231 | 11 | 242 |
|  |  | % within Sex | 95.5% | 4.5% | 100.0% |

|  | | | Maxillary sinusitis | | Total |
| --- | --- | --- | --- | --- | --- |
|  |  |  | 0 | 1 |  |
| Sex | F | Count | 19 | 24 | 43 |
|  |  | % within Sex | 44.2% | 55.8% | 100.0% |
|  | M | Count | 36 | 53 | 89 |
|  |  | % within Sex | 40.4% | 59.6% | 100.0% |
| Total | | Count | 55 | 77 | 132 |
|  |  | % within Sex | 41.7% | 58.3% | 100.0% |

|  | | | Infectious disease | | Total |
| --- | --- | --- | --- | --- | --- |
|  |  |  | 0 | 1 |  |
| Sex | F | Count | 85 | 20 | 105 |
|  |  | % within Sex | 81.0% | 19.0% | 100.0% |
|  | M | Count | 136 | 9 | 145 |
|  |  | % within Sex | 93.8% | 6.2% | 100.0% |
| Total | | Count | 221 | 29 | 250 |
|  |  | % within Sex | 88.4% | 11.6% | 100.0% |

|  | | | TB or respiratory condition | | Total |
| --- | --- | --- | --- | --- | --- |
|  |  |  | 0 | 1 |  |
| Sex | F | Count | 31 | 27 | 58 |
|  |  | % within Sex | 53.4% | 46.6% | 100.0% |
|  | M | Count | 46 | 33 | 79 |
|  |  | % within Sex | 58.2% | 41.8% | 100.0% |
| Total | | Count | 77 | 60 | 137 |
|  |  | % within Sex | 56.2% | 43.8% | 100.0% |

|  | | | TB | | Total |
| --- | --- | --- | --- | --- | --- |
|  |  |  | .00 | 1.00 |  |
| Sex | F | Count | 73 | 19 | 92 |
|  |  | % within Sex | 79.3% | 20.7% | 100.0% |
|  | M | Count | 120 | 11 | 131 |
|  |  | % within Sex | 91.6% | 8.4% | 100.0% |
| Total | | Count | 193 | 30 | 223 |
|  |  | % within Sex | 86.5% | 13.5% | 100.0% |

|  | | | DISH | | Total |
| --- | --- | --- | --- | --- | --- |
|  |  |  | 0 | 1 |  |
| Sex | F | Count | 74 | 2 | 76 |
|  |  | % within Sex | 97.4% | 2.6% | 100.0% |
|  | M | Count | 96 | 20 | 116 |
|  |  | % within Sex | 82.8% | 17.2% | 100.0% |
| Total | | Count | 170 | 22 | 192 |
|  |  | % within Sex | 88.5% | 11.5% | 100.0% |

|  | | | Hallux valgus | | Total |
| --- | --- | --- | --- | --- | --- |
|  |  |  | 0 | 1 |  |
| Sex | F | Count | 33 | 5 | 38 |
|  |  | % within Sex | 86.8% | 13.2% | 100.0% |
|  | M | Count | 50 | 21 | 71 |
|  |  | % within Sex | 70.4% | 29.6% | 100.0% |
| Total | | Count | 83 | 26 | 109 |
|  |  | % within Sex | 76.1% | 23.9% | 100.0% |

|  | | | Extra spinal OA | | Total |
| --- | --- | --- | --- | --- | --- |
|  |  |  | 0 | 1 |  |
| Sex | F | Count | 67 | 37 | 104 |
|  |  | % within Sex | 64.4% | 35.6% | 100.0% |
|  | M | Count | 88 | 64 | 152 |
|  |  | % within Sex | 57.9% | 42.1% | 100.0% |
| Total | | Count | 155 | 101 | 256 |
|  |  | % within Sex | 60.5% | 39.5% | 100.0% |

|  | | | Trauma | | Total |
| --- | --- | --- | --- | --- | --- |
|  |  |  | 0 | 1 |  |
| Sex | F | Count | 80 | 31 | 111 |
|  |  | % within Sex | 72.1% | 27.9% | 100.0% |
|  | M | Count | 97 | 67 | 164 |
|  |  | % within Sex | 59.1% | 40.9% | 100.0% |
| Total | | Count | 177 | 98 | 275 |
|  |  | % within Sex | 64.4% | 35.6% | 100.0% |

|  | | | Cranial trauma | | Total |
| --- | --- | --- | --- | --- | --- |
|  |  |  | 0 | 1 |  |
| Sex | F | Count | 60 | 4 | 64 |
|  |  | % within Sex | 93.8% | 6.3% | 100.0% |
|  | M | Count | 113 | 9 | 122 |
|  |  | % within Sex | 92.6% | 7.4% | 100.0% |
| Total | | Count | 173 | 13 | 186 |
|  |  | % within Sex | 93.0% | 7.0% | 100.0% |

|  | | | Schmorl’s nodes (number per person) | | | Total |
| --- | --- | --- | --- | --- | --- | --- |
|  |  |  | 1-6 | 7+ | none |  |
| Sex | F | Count | 28 | 7 | 44 | 79 |
|  |  | % within Sex | 35.4% | 8.9% | 55.7% | 100.0% |
|  | M | Count | 47 | 19 | 45 | 111 |
|  |  | % within Sex | 42.3% | 17.1% | 40.5% | 100.0% |
| Total | | Count | 75 | 26 | 89 | 190 |
|  |  | % within Sex | 39.5% | 13.7% | 46.8% | 100.0% |

|  | | | Adult age at death | | | Total |
| --- | --- | --- | --- | --- | --- | --- |
|  |  |  | 18-25 | 26-45 | 45- |  |
| Sex | F | Count | 20 | 46 | 54 | 120 |
|  |  | % within Sex | 16.7% | 38.3% | 45.0% | 100.0% |
|  | M | Count | 27 | 83 | 78 | 188 |
|  |  | % within Sex | 14.4% | 44.1% | 41.5% | 100.0% |
| Total | | Count | 47 | 129 | 132 | 308 |
|  |  | % within Sex | 15.3% | 41.9% | 42.9% | 100.0% |

| Sex | | Stature | Dentine mean d13C | Rib mean d13C | Dentine mean d15N |
| --- | --- | --- | --- | --- | --- |
| F | Mean | 161.39645 | -19.351 | -19.2466 | 11.960 |
|  | N | 108 | 47 | 58 | 47 |
|  | Std. Deviation | 5.541688 | .4432 | .38807 | 1.0458 |
| M | Mean | 170.22609 | -19.162 | -18.9063 | 12.074 |
|  | N | 163 | 69 | 111 | 69 |
|  | Std. Deviation | 5.049242 | .4624 | .47850 | 1.3834 |
| Total | Mean | 166.70727 | -19.239 | -19.0231 | 12.028 |
|  | N | 271 | 116 | 169 | 116 |
|  | Std. Deviation | 6.798847 | .4623 | .47672 | 1.2539 |

| Sex | | Rib mean d15N |
| --- | --- | --- |
| F | Mean | 12.288 |
|  | N | 58 |
|  | Std. Deviation | .9287 |
| M | Mean | 12.868 |
|  | N | 111 |
|  | Std. Deviation | 1.1489 |
| Total | Mean | 12.669 |
|  | N | 169 |
|  | Std. Deviation | 1.1106 |

**Supplementary Information 3. Distribution of all indicators by social group**

|  | | | More than one hypoplastic lesion | | Total |
| --- | --- | --- | --- | --- | --- |
|  |  |  | .00 | 1.00 |  |
| Social Group | Charity | Count | 20 | 43 | 63 |
|  |  | % within Social Group | 31.7% | 68.3% | 100.0% |
|  | Friars | Count | 5 | 11 | 16 |
|  |  | % within Social Group | 31.3% | 68.8% | 100.0% |
|  | Lay | Count | 28 | 27 | 55 |
|  |  | % within Social Group | 50.9% | 49.1% | 100.0% |
| Total | | Count | 53 | 81 | 134 |
|  |  | % within Social Group | 39.6% | 60.4% | 100.0% |

|  | | | Cribra orbitalia | | Total |
| --- | --- | --- | --- | --- | --- |
|  |  |  | 0 | 1 |  |
| Social Group | Charity | Count | 31 | 37 | 68 |
|  |  | % within Social Group | 45.6% | 54.4% | 100.0% |
|  | Friars | Count | 11 | 4 | 15 |
|  |  | % within Social Group | 73.3% | 26.7% | 100.0% |
|  | Lay | Count | 33 | 21 | 54 |
|  |  | % within Social Group | 61.1% | 38.9% | 100.0% |
| Total | | Count | 75 | 62 | 137 |
|  |  | % within Social Group | 54.7% | 45.3% | 100.0% |

|  | | | Vitamin D deficiency | | Total |
| --- | --- | --- | --- | --- | --- |
|  |  |  | 0 | 1 |  |
| Social Group | Charity | Count | 153 | 10 | 163 |
|  |  | % within Social Group | 93.9% | 6.1% | 100.0% |
|  | Friars | Count | 17 | 0 | 17 |
|  |  | % within Social Group | 100.0% | 0.0% | 100.0% |
|  | Lay | Count | 101 | 1 | 102 |
|  |  | % within Social Group | 99.0% | 1.0% | 100.0% |
| Total | | Count | 271 | 11 | 282 |
|  |  | % within Social Group | 96.1% | 3.9% | 100.0% |

|  | | | Maxillary sinusitis | | Total |
| --- | --- | --- | --- | --- | --- |
|  |  |  | 0 | 1 |  |
| Social Group | Charity | Count | 20 | 40 | 60 |
|  |  | % within Social Group | 33.3% | 66.7% | 100.0% |
|  | Friars | Count | 4 | 11 | 15 |
|  |  | % within Social Group | 26.7% | 73.3% | 100.0% |
|  | Lay | Count | 22 | 17 | 39 |
|  |  | % within Social Group | 56.4% | 43.6% | 100.0% |
| Total | | Count | 46 | 68 | 114 |
|  |  | % within Social Group | 40.4% | 59.6% | 100.0% |

|  | | | Infectious disease | | Total |
| --- | --- | --- | --- | --- | --- |
|  |  |  | 0 | 1 |  |
| Social Group | Charity | Count | 144 | 22 | 166 |
|  |  | % within Social Group | 86.7% | 13.3% | 100.0% |
|  | Friars | Count | 15 | 0 | 15 |
|  |  | % within Social Group | 100.0% | 0.0% | 100.0% |
|  | Lay | Count | 89 | 8 | 97 |
|  |  | % within Social Group | 91.8% | 8.2% | 100.0% |
| Total | | Count | 248 | 30 | 278 |
|  |  | % within Social Group | 89.2% | 10.8% | 100.0% |

|  | | | TB or respiratory condition | | Total |
| --- | --- | --- | --- | --- | --- |
|  |  |  | 0 | 1 |  |
| Social Group | Charity | Count | 60 | 38 | 98 |
|  |  | % within Social Group | 61.2% | 38.8% | 100.0% |
|  | Friars | Count | 2 | 6 | 8 |
|  |  | % within Social Group | 25.0% | 75.0% | 100.0% |
|  | Lay | Count | 19 | 19 | 38 |
|  |  | % within Social Group | 50.0% | 50.0% | 100.0% |
| Total | | Count | 81 | 63 | 144 |
|  |  | % within Social Group | 56.3% | 43.8% | 100.0% |

|  | | | TB | | Total |
| --- | --- | --- | --- | --- | --- |
|  |  |  | .00 | 1.00 |  |
| Social Group | Charity | Count | 113 | 21 | 134 |
|  |  | % within Social Group | 84.3% | 15.7% | 100.0% |
|  | Friars | Count | 15 | 2 | 17 |
|  |  | % within Social Group | 88.2% | 11.8% | 100.0% |
|  | Lay | Count | 74 | 8 | 82 |
|  |  | % within Social Group | 90.2% | 9.8% | 100.0% |
| Total | | Count | 202 | 31 | 233 |
|  |  | % within Social Group | 86.7% | 13.3% | 100.0% |

|  | | | DISH | | Total |
| --- | --- | --- | --- | --- | --- |
|  |  |  | 0 | 1 |  |
| Social Group | Charity | Count | 93 | 16 | 109 |
|  |  | % within Social Group | 85.3% | 14.7% | 100.0% |
|  | Friars | Count | 16 | 2 | 18 |
|  |  | % within Social Group | 88.9% | 11.1% | 100.0% |
|  | Lay | Count | 69 | 4 | 73 |
|  |  | % within Social Group | 94.5% | 5.5% | 100.0% |
| Total | | Count | 178 | 22 | 200 |
|  |  | % within Social Group | 89.0% | 11.0% | 100.0% |

|  | | | Hallux valgus | | Total |
| --- | --- | --- | --- | --- | --- |
|  |  |  | 0 | 1 |  |
| Social Group | Charity | Count | 62 | 16 | 78 |
|  |  | % within Social Group | 79.5% | 20.5% | 100.0% |
|  | Friars | Count | 6 | 5 | 11 |
|  |  | % within Social Group | 54.5% | 45.5% | 100.0% |
|  | Lay | Count | 46 | 9 | 55 |
|  |  | % within Social Group | 83.6% | 16.4% | 100.0% |
| Total | | Count | 114 | 30 | 144 |
|  |  | % within Social Group | 79.2% | 20.8% | 100.0% |

|  | | | Extra spinal OA | | Total |
| --- | --- | --- | --- | --- | --- |
|  |  |  | 0 | 1 |  |
| Social Group | Charity | Count | 120 | 47 | 167 |
|  |  | % within Social Group | 71.9% | 28.1% | 100.0% |
|  | Friars | Count | 7 | 11 | 18 |
|  |  | % within Social Group | 38.9% | 61.1% | 100.0% |
|  | Lay | Count | 55 | 52 | 107 |
|  |  | % within Social Group | 51.4% | 48.6% | 100.0% |
| Total | | Count | 182 | 110 | 292 |
|  |  | % within Social Group | 62.3% | 37.7% | 100.0% |

|  | | | Trauma | | Total |
| --- | --- | --- | --- | --- | --- |
|  |  |  | 0 | 1 |  |
| Social Group | Charity | Count | 134 | 51 | 185 |
|  |  | % within Social Group | 72.4% | 27.6% | 100.0% |
|  | Friars | Count | 11 | 6 | 17 |
|  |  | % within Social Group | 64.7% | 35.3% | 100.0% |
|  | Lay | Count | 72 | 47 | 119 |
|  |  | % within Social Group | 60.5% | 39.5% | 100.0% |
| Total | | Count | 217 | 104 | 321 |
|  |  | % within Social Group | 67.6% | 32.4% | 100.0% |

|  | | | Cranial trauma | | Total |
| --- | --- | --- | --- | --- | --- |
|  |  |  | 0 | 1 |  |
| Social Group | Charity | Count | 64 | 5 | 69 |
|  |  | % within Social Group | 92.8% | 7.2% | 100.0% |
|  | Friars | Count | 12 | 1 | 13 |
|  |  | % within Social Group | 92.3% | 7.7% | 100.0% |
|  | Lay | Count | 57 | 6 | 63 |
|  |  | % within Social Group | 90.5% | 9.5% | 100.0% |
| Total | | Count | 133 | 12 | 145 |
|  |  | % within Social Group | 91.7% | 8.3% | 100.0% |

|  | | | Schmorl’s nodes (number per person) | | | Total |
| --- | --- | --- | --- | --- | --- | --- |
|  |  |  | 1-6 | 7+ | none |  |
| Social Group | Charity | Count | 51 | 12 | 48 | 111 |
|  |  | % within Social Group | 45.9% | 10.8% | 43.2% | 100.0% |
|  | Friars | Count | 7 | 5 | 5 | 17 |
|  |  | % within Social Group | 41.2% | 29.4% | 29.4% | 100.0% |
|  | Lay | Count | 19 | 10 | 40 | 69 |
|  |  | % within Social Group | 27.5% | 14.5% | 58.0% | 100.0% |
| Total | | Count | 77 | 27 | 93 | 197 |
|  |  | % within Social Group | 39.1% | 13.7% | 47.2% | 100.0% |

|  | | | Adult age at death | | | Total |
| --- | --- | --- | --- | --- | --- | --- |
|  |  |  | 18-25 | 26-45 | 45- |  |
| Social Group | Charity | Count | 43 | 58 | 62 | 163 |
|  |  | % within Social Group | 26.4% | 35.6% | 38.0% | 100.0% |
|  | Friars | Count | 4 | 7 | 7 | 18 |
|  |  | % within Social Group | 22.2% | 38.9% | 38.9% | 100.0% |
|  | Lay | Count | 16 | 69 | 64 | 149 |
|  |  | % within Social Group | 10.7% | 46.3% | 43.0% | 100.0% |
| Total | | Count | 63 | 134 | 133 | 330 |
|  |  | % within Social Group | 19.1% | 40.6% | 40.3% | 100.0% |

| Stature | | | | |
| --- | --- | --- | --- | --- |
| Sex | Social Group | Mean | N | Std. Deviation |
| F | Charity | 160.23667 | 49 | 5.877228 |
|  | Lay | 162.35966 | 59 | 5.097918 |
|  | Total | 161.39645 | 108 | 5.541688 |
| M | Charity | 169.14643 | 63 | 4.446292 |
|  | Friars | 173.43135 | 17 | 4.957317 |
|  | Lay | 170.37411 | 64 | 5.471272 |
|  | Total | 170.19792 | 144 | 5.123770 |
| Total | Charity | 165.24841 | 112 | 6.759532 |
|  | Friars | 173.43135 | 17 | 4.957317 |
|  | Lay | 166.52978 | 123 | 6.631453 |
|  | Total | 166.42587 | 252 | 6.862606 |

| Social Group | | Dentine mean d13C | Rib mean d13C | Dentine mean d15N | Rib mean d15N |
| --- | --- | --- | --- | --- | --- |
| Charity | Mean | -19.162 | -19.0216 | 12.079 | 12.475 |
|  | N | 56 | 116 | 56 | 116 |
|  | Std. Deviation | .4960 | .40061 | 1.3449 | 1.1346 |
| Friars | Mean | -18.975 | -18.4176 | 12.400 | 13.929 |
|  | N | 12 | 17 | 12 | 17 |
|  | Std. Deviation | .4202 | .38444 | 1.0260 | .4985 |
| Lay | Mean | -19.388 | -19.2674 | 11.904 | 12.589 |
|  | N | 49 | 46 | 49 | 46 |
|  | Std. Deviation | .3784 | .47190 | 1.1974 | .8777 |
| Total | Mean | -19.238 | -19.0274 | 12.038 | 12.642 |
|  | N | 117 | 179 | 117 | 179 |
|  | Std. Deviation | .4605 | .47295 | 1.2540 | 1.1072 |

**Supplementary Information 4. Graphic representations of all data**

| 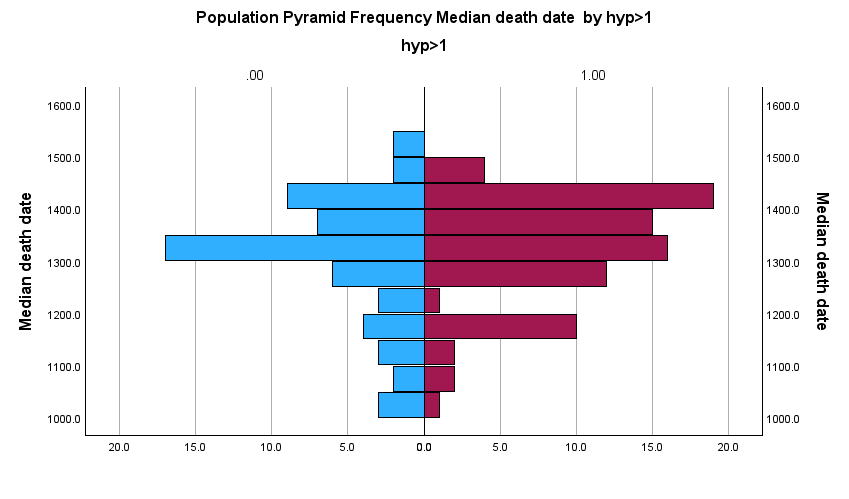 | 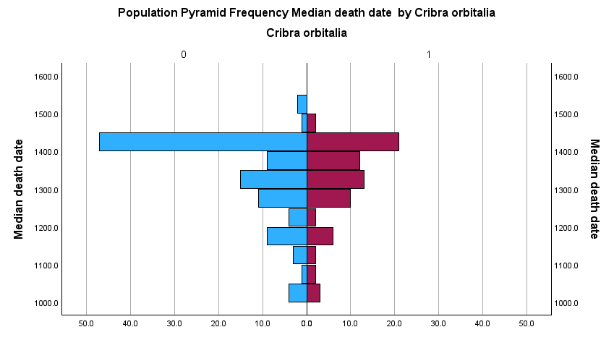 |
| --- | --- |
| 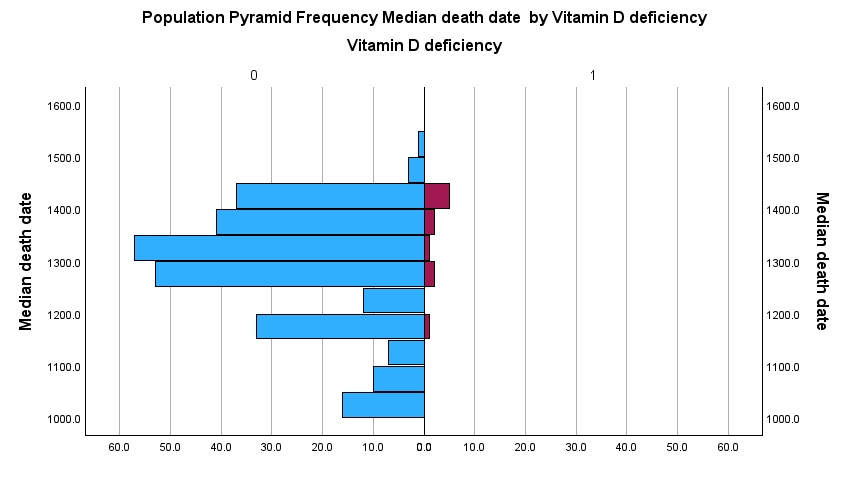 | 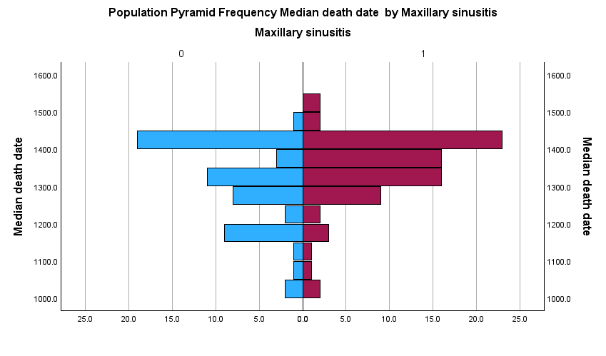 |
| 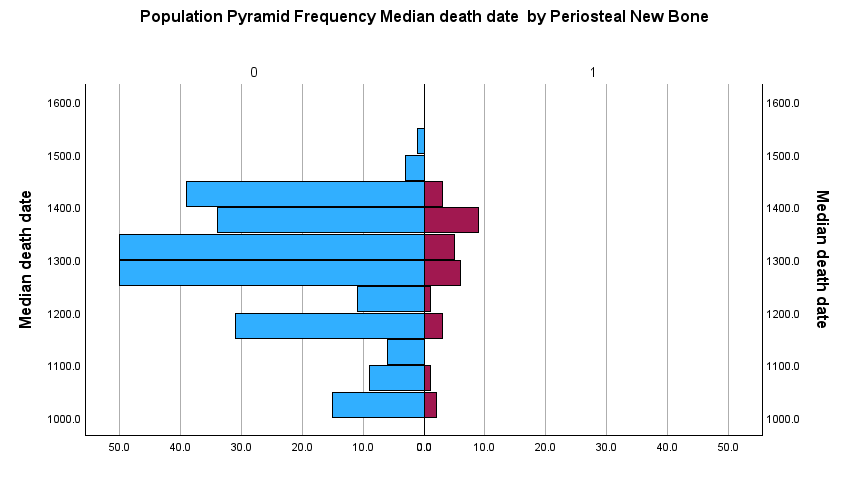 | 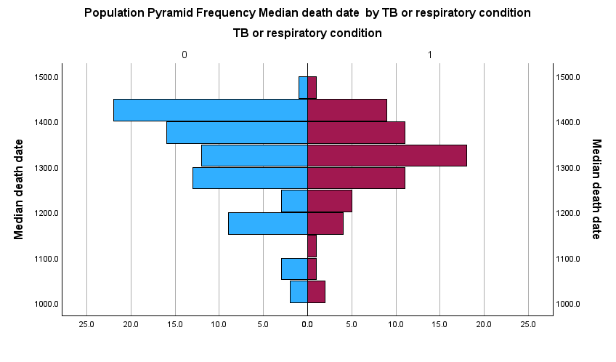 |
| 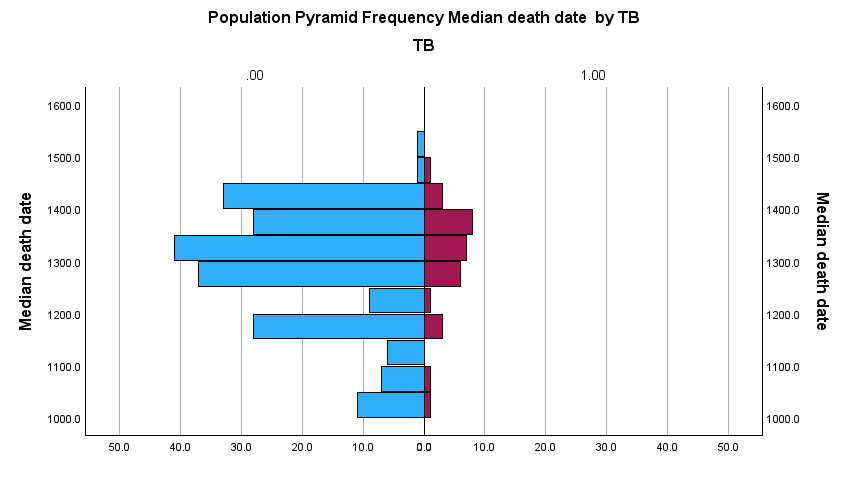 | 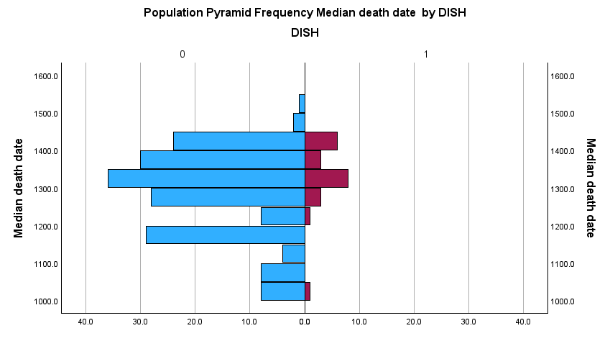 |
| 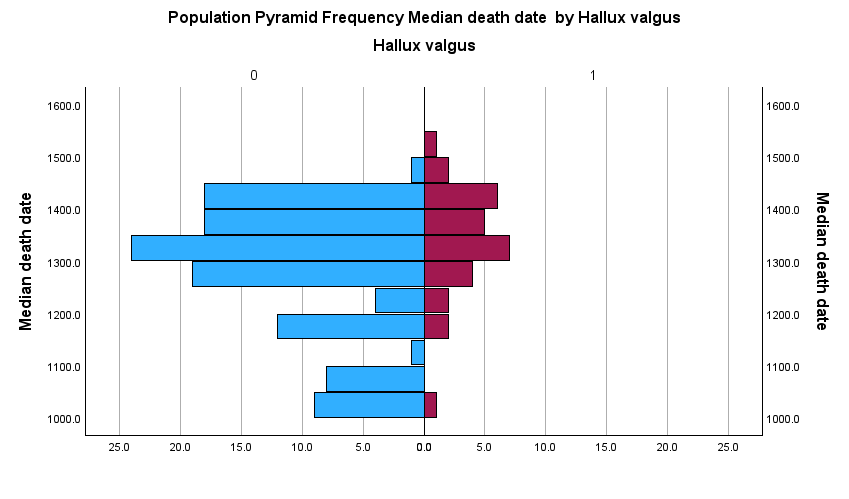 | 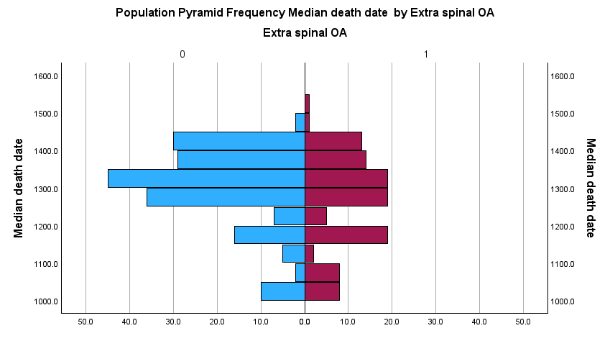 |
| 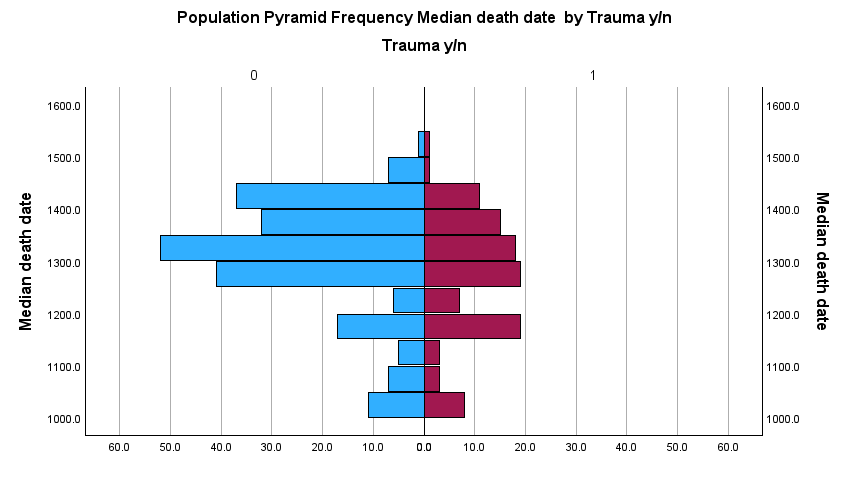 | 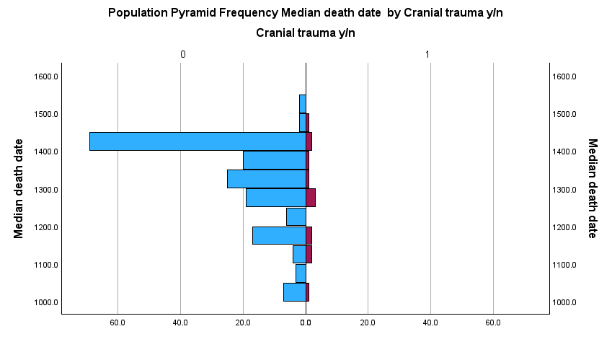 |
| 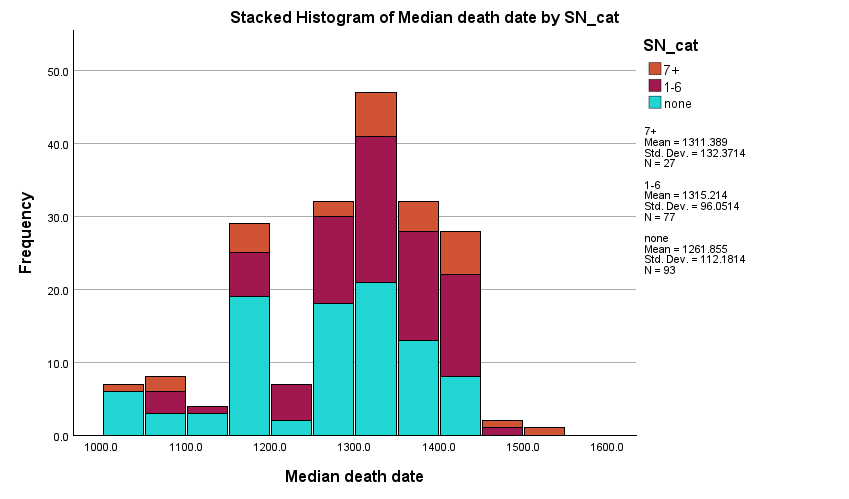 | 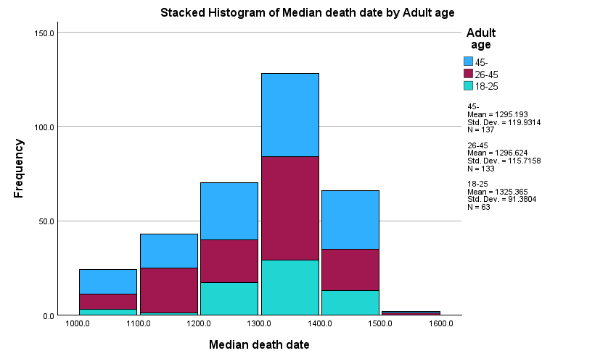 |
| 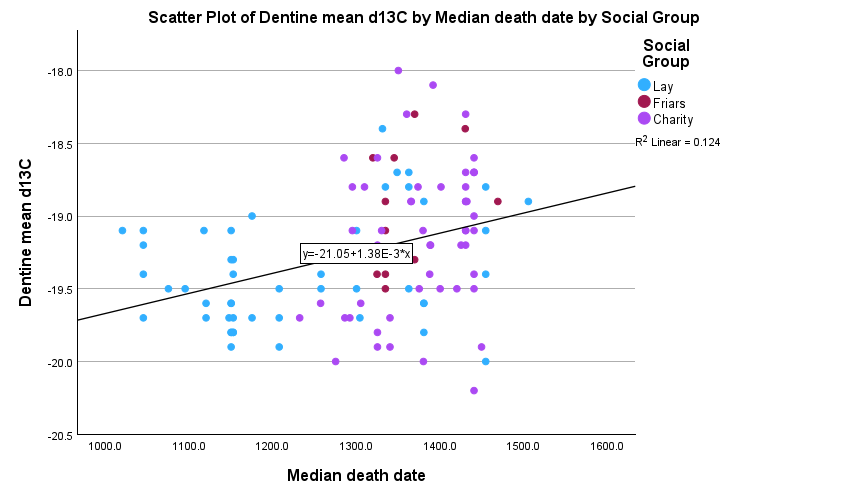 | 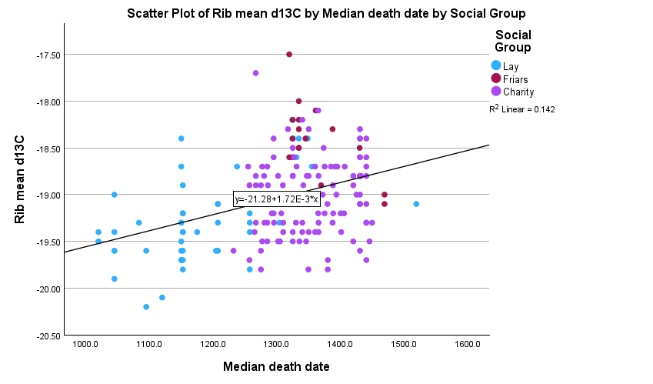 |
| 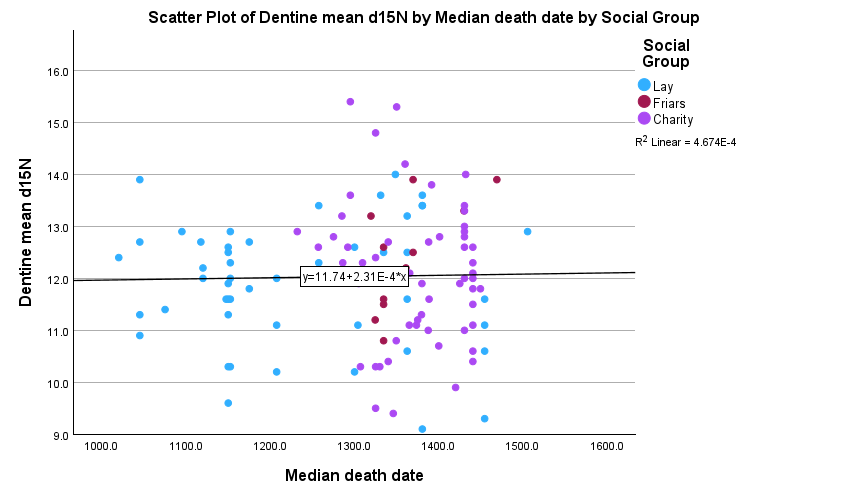 | 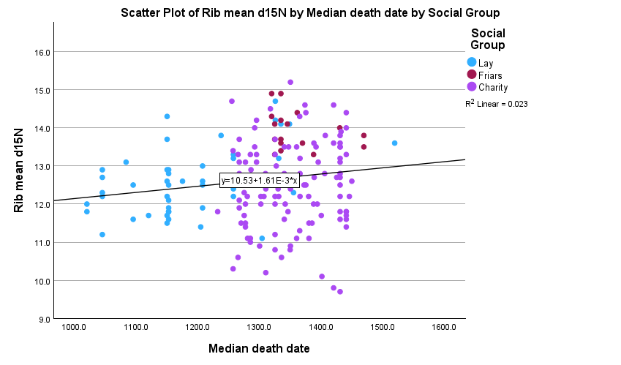 |
| 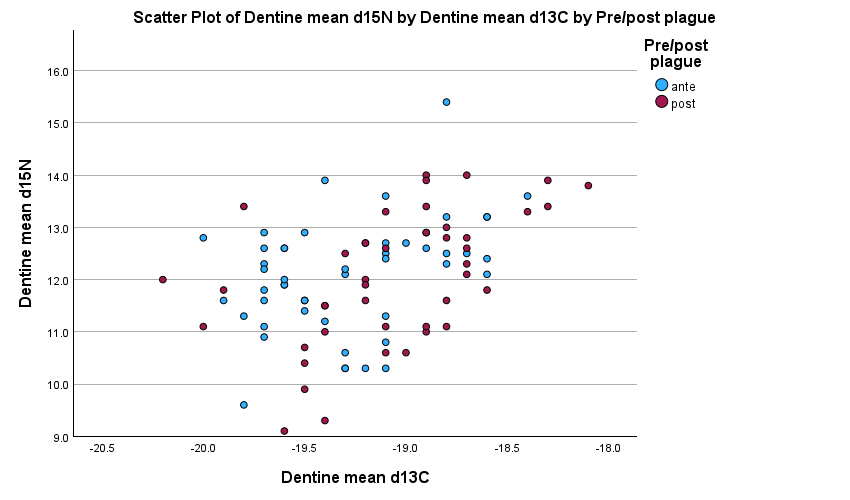 | 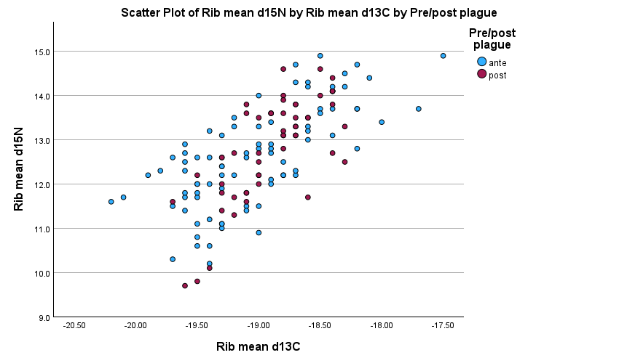 |
| 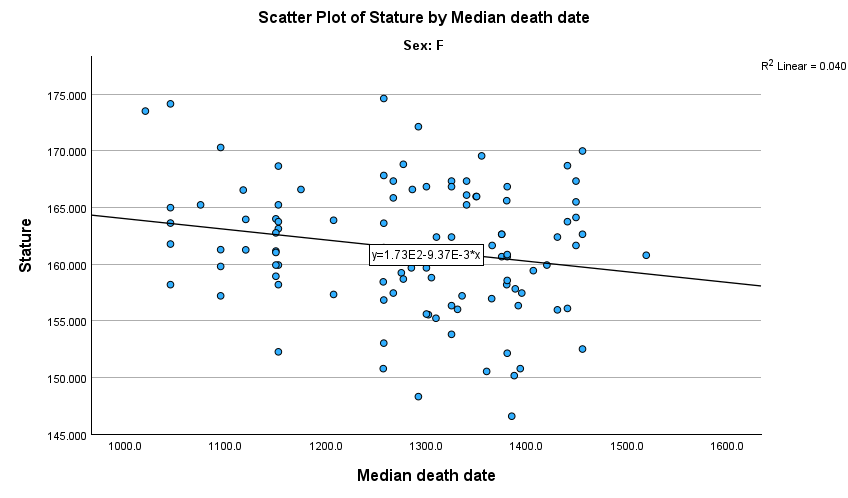 | 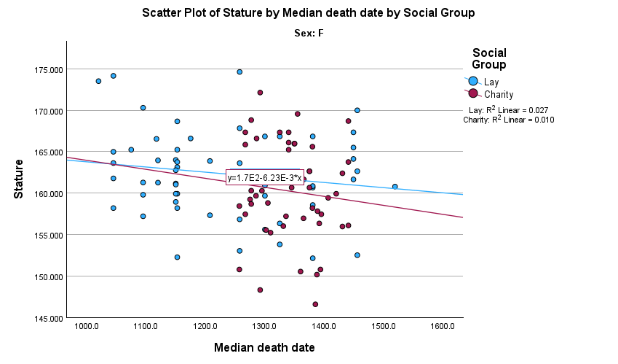 |
| 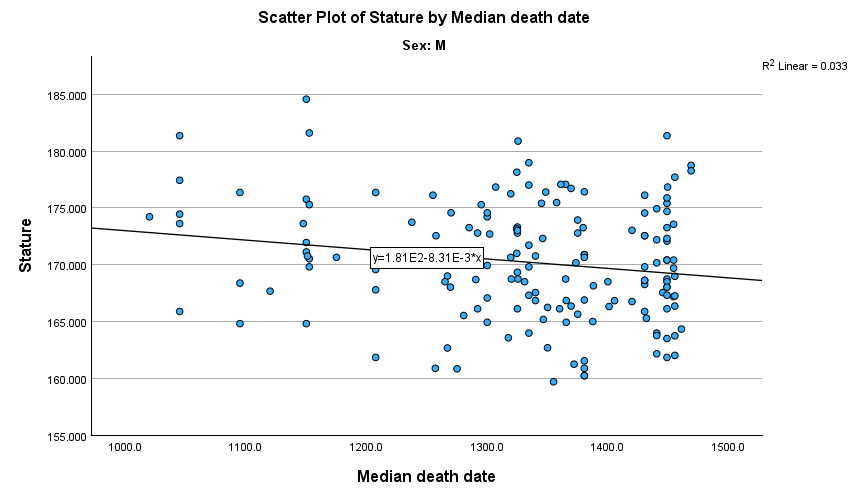 | 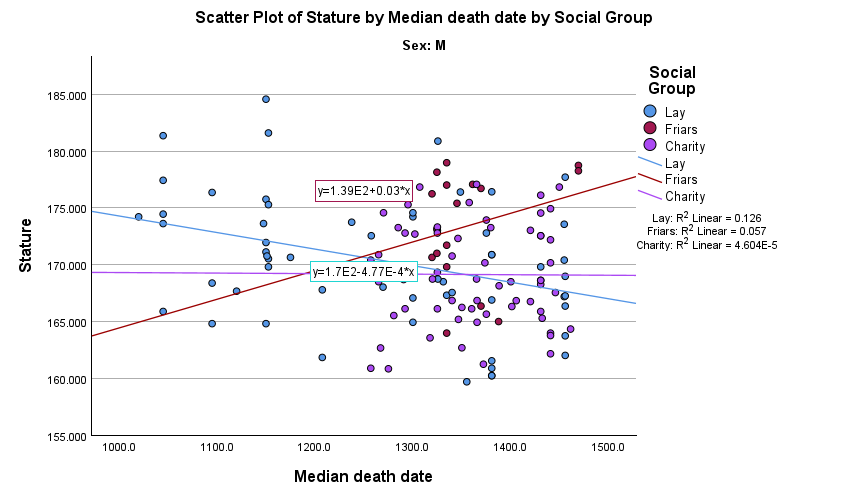 |
